# Problematic social media use, mental health, and substance use in Czech adolescents: the comparative role of extracurricular activities — findings from the 2022 HBSC study

**DOI:** 10.1186/s13690-026-01993-1

**Published:** 2026-06-16

**Authors:** Jakub Helvich, Vendula Machu, Lukas Novak, Barbora Kuta, Zdenek Meier, Petr Badura, Peter Tavel

**Affiliations:** 1 Olomouc University Social Health Institute, Palacky University in Olomouc, Olomouc, Czech Republic; 2 Department of Health Sciences, Community and Occupational Medicine, University Medical Center Groningen, University of Groningen, Groningen, The Netherlands

**Keywords:** Problematic social media use, Mental health, Extracurricular activities, Network analysis, HBSC

## Abstract

**Background:**

Problematic social media use (PSMU) has become a critical public health concern. However, little is known about how the associations between PSMU, mental health, and substance use differ depending on participation in distinct types of extracurricular activities.

**Objective:**

The objective of this study is to examine how the associations between PSMU, mental health, and substance use vary across network models that include different types of extracurricular activities.

**Methodology:**

A representative sample of 14,588 Czech adolescents (50.69% boys, mean age 13.6 ± 1.7 years) was used from the 2022 Health Behaviour in School-aged Children study. A network analysis based on undirected mixed graphical models was used to assess the associations. Separate networks for each extracurricular activity type were estimated and compared.

**Results:**

When individual sports were included in the network, the association between alcohol use and PSMU was no longer observed. In both the community-based activities and individual sports networks, the previously observed positive association between irritability and PSMU-related family conflicts (w = 0.05) was absent, and the association between irritability and social media escapism was weaker (w = 0.08 → 0.03). Only sports-related activity types were negatively associated with losing interest in extracurricular activities due to PSMU, particularly individual sports (w = − 0.10). Conversely, community-based activities were positively associated with PSMU-related deception and family conflicts (w = 0.05). However, no notable differences in the network structure were observed for artistic activities or team sports.

**Conclusion:**

The study underscores the contrastive nature of extracurricular activities and highlights the need for more nuanced approaches when examining their role in PSMU.

**Supplementary Information:**

The online version contains supplementary material available at 10.1186/s13690-026-01993-1.

**Table:** 

Text box 1. Contributions to the literature	
• Most public health studies examine problematic social media use using overall scores; this study advances the field by applying symptom-level network analysis in a large, nationally representative adolescent sample. • It provides the first direct comparison of how the associations between problematic social media behaviours, mental health symptoms, and substance use vary across participation in distinct types of extracurricular activities.	
• The findings show that extracurricular activities do not function uniformly: individual and team sports, community-based activities, and arts relate to changes between problematic social media use correlates in distinct ways. • By identifying activity-specific patterns, the study informs more targeted population-level and youth health promotion strategies.	

## Background

Problematic social media use (PSMU) refers to maladaptive use of social media platforms that exhibit signs of addiction-like behaviours, such as loss of control, withdrawal symptoms, and changes in tolerance, leading to impairments in daily functioning and well-being [1, 2]. In the past decade, PSMU has become a critical public health issue, with evidence showing a significantly co-occurrence with negative health outcomes. It is particularly evident among adolescents, who are navigating a formative stage of emotional, cognitive, and social development [3–5]. Given that adolescents represent one of the most active groups on social media platforms [5, 6], the prevalence of PSMU is especially concerning.

In the U.S., about 70% of adolescents have at least one social media account [7]. Even higher numbers are reported in the UK, where about 93% of adolescents have a social media profile [8]. An international HBSC survey of adolescents found that about 78% were classified as active or intense social media users [9]. Consequently, this surge has also led to an increase in PSMU. In 2022, 11% of adolescents across 44 European and Central Asian countries showed signs of PSMU, up from 7% in 2018 [3, 10]. The Czech Republic shows similarly pervasive PSMU among adolescents. A representative Czech study reported that 25.9% of adolescents spent over 2 h per day on social media [11] and about 8% of adolescents (ages 11–15) were classified as PSMU users in 2022, an increase from 5% in 2018 [3, 12]. Altogether, this underscores an alarming global trend, which is especially concerning since PSMU may have far-reaching consequences on youths’ health.

Research has repeatedly linked PSMU to a wide range of mental health symptoms. PSMU has been consistently associated with depressed mood, increased stress and anxiety levels [13–16]. Additionally, previous studies revealed that adolescents who spend excessive time on social media often experience poorer sleep quality [17]. Sleep disturbances, then in turn, may contribute to fatigue, irritability, and low mood, further reinforcing the mental health issues [18–20]. Moreover, cognitive impairments, such as reduced attention span, memory deficits, and decreased executive function, have also been linked to PSMU [17, 21, 22]. Recent evidence also shows positive links between PSMU and substance use, including alcohol, cannabis, and tobacco [23–25], with younger individuals, in particular, being at-risk [26]. Nevertheless, despite these long-standing findings, critical gaps remain. Specifically, little is known about how the associations between PSMU, mental health, and substance use may differ depending on participation in distinct types of extracurricular activities (e.g., team sports, individual sports, artistic pursuits, etc.).

Participation in extracurricular activities has been linked to better adolescent mental health, including higher life satisfaction and lower anxiety and depressive symptoms [27, 28]. Research also suggests that adolescents involved in extracurricular activities tend to have lower screen time [26, 29]. Conversely, adolescents with no extracurricular engagements may have more unstructured free time that they end up devoting to social media, potentially to a detrimental means [26]. Additionally, extracurricular activities may offer alternative outlets for coping and human connection that may reduce reliance on social media and health-risk substances [29]. Organised sports in particular are associated with higher psychological well-being, with participants having lower rates of depression and anxiety and better stress regulation [29, 30].

On the contrary, participation in artistic activities was shown to be associated with higher levels of depressive symptoms and anxiety in adolescents and young adults [31]. Previously, it has been suggested that adolescents engaged in artistic activities may differ in underlying cognitive characteristics [32], which could play a role in how PSMU relates to their mental health. In contrast, participating in community-based activities builds social skills, a sense of belonging and empathy, which may alleviate negative feelings and can increase the sense of social connection and sense of purpose [33, 34]. Altogether, we expect that the associations between problematic social media use and mental health symptoms would be weaker among adolescents participating in organised sports and community‑based activities, and more pronounced among those engaged in artistic activities. The limited findings of previous studies point to the potential associations between extracurricular participation, better mental health and decreasing PSMU. However, several critical research gaps remain unexplored and unresolved.

To date, no research has utilized network analysis to simultaneously model and contrast participation in various types of extracurricular activities or lacked sufficiently representative sample size [27, 28, 35], failing to compare their differential role in the associations between PSMU, mental health, and substance use. As a result, any activity-specific patterns remain largely unexplored. Moreover, there is limited evidence of how distinct types of extracurricular activities are associated with changes in the item-level connections between mental health symptoms, PSMU symptoms, and various health-risk substances.

Therefore, this study aims to use network analysis to describe and compare the patterns of associations between PSMU, mental health symptoms, and substance use across four groups of Czech adolescents: those participating in team sports, individual sports, artistic activities, and community-based activities.

## Methods

### Participants and procedure

A nationally representative dataset of Czech adolescents was used from the 2022 Health Behaviour in School-aged Children (HBSC) study. Data collection took place between May and June 2022, following a standardised, internationally developed research protocol [36] available at: https://www.hbsc.org/publications/survey-protocols. Schools were chosen using a randomised selection process, considering the principles of probability-proportional-to-size based region and school type (primary or secondary). Of the 272 schools contacted, 246 agreed to participate, resulting in a response rate of 90.4%. Within each participating school, a single class was randomly selected from grades 5, 7, and 9, representing pupils aged 11, 13, and 15, yielding a total sample of 14,879 pupils. Participation was fully anonymous and entirely voluntary. The overall pupil response rate was 83.1%. Most non-responses were attributed to illness (*n* = 1928), engagement in academic or athletic events (*n* = 1024), or voluntary withdrawal (*n* = 77).

During the data processing procedure, an additional number of entries (*n* = 111) were excluded due to logical inconsistencies, such as contradictory responses or implausible entries in open-ended items. Further records (*n* = 12) were removed due to excessive missing data. Additionally, a total of *n* = 168 respondents were removed from the sample due to age outside the permitted range. The final dataset used for analysis included *n* = 14,588 respondents, comprising 50.69% boys, with a mean age of 13.6 ± 1.7 years.

The study received ethical clearance from the Ethics Committee of the Faculty of Physical Culture, Palacký University Olomouc (Approval No. 14/2019) and complied with the principles of the Convention on Human Rights and Biomedicine (40/2000 Coll.).

### Measures

*The Social Media Disorder Scale (SMDS)* is a brief self-report instrument designed to gauge a range of indicators indicative of PSMU [37]. Comprising nine dichotomous (yes/no) items, the scale covers core disorder-related behaviours, such as preoccupation with social media, escalating use, emotional withdrawal when access is restricted, and disruptions to personal relationships. In our study, we used the validated Czech version of the SMDS [38]. Respondents rate their experiences with social media use over the past 12 months. Total scores range from 0 to 9, with higher totals indicating more severe PSMU. For the present study, these nine items were treated as independent nodes in the network analysis rather than being aggregated into a single composite score. Retaining the individual items allows for a highly granular examination of how specific characteristics of PSMU differentially connect to specific mental health symptoms, substance use behaviors, and extracurricular activities.

To complement the SMDS, the study also included an additional item examining emotional expression preferences. Specifically, participants were asked whether they are more comfortable sharing their feelings through social media rather than in face-to-face interactions. Responses were recorded on a five-point Likert scale, ranging from 1 (“Strongly Disagree”) to 5 (“Strongly Agree”) and the item was treated as continuous.

*The Multiple Health Complaints Measure* is a self-administered tool designed to capture common psychosomatic and emotional symptoms [39]. It comprises eight items, divided equally between physical complaints (e.g., headaches, stomachaches, back pain, and dizziness) and psychological symptoms (e.g., feeling down, nervousness, irritability, and sleep difficulties). For this study, only the psychological items were utilised. Somatic complaints such as headaches or stomachaches may arise from a broader range of physiological or contextual factors that are not necessarily related to social media behaviors and excluding physical symptoms therefore helped maintain conceptual specificity in the subsequent analysis. Participants reported the frequency of these experiences over the previous six months using a five-point scale ranging from 1 (“About every day”) to 5 (“Rarely or never”). For the present study, the items were treated as independent continuous variables in the network analysis. The item scoring was reversed so that higher values reflect more frequent symptoms. This reverse scoring was implemented prior to analysis to ensure that higher values consistently reflect higher symptom severity as indicated by construct phrasing to improve subsequent interpretability of findings.

*The World Health Organisation-Five Well-Being Index* (*WHO-5)* is a brief self-report measure designed to assess psychological well-being [40]. It consists of five positively framed statements capturing aspects such as mood, energy levels, and interest in daily activities, based on experiences over the past two weeks. Respondents rate each item on a six-point scale, from 0 (“at no time”) to 5 (“all of the time”) and the individual items were treated as continuous in the analysis.

*Substance use* in this study was assessed using three items addressing substance-related risk-taking behaviours. Respondents were asked: “*How many days (if at all) have you [item] in the past 30 days?*”, with items: (1) smoked cigarettes, (2) used electronic cigarettes (excluding heated tobacco), or (3) drunk alcohol. Each item is then rated on a 7-point scale from 1 (“never ”) to 7 (“30 days or more”) and each item was treated as ordinal. A higher score indicates a more frequent substance use.

*The Family Affluence Scale (FAS)* serves as an indicator of adolescents’ material living standards and family socioeconomic background [41]. It assesses household resources and amenities, such as car ownership, availability of a dishwasher, number of bathrooms and computers, whether the child has their own bedroom, and the frequency of family holidays abroad. The total score ranges from 0 to 13, with higher scores indicating higher socioeconomic status.

*Extracurricular activities* were grouped into 4 categories, with each focusing on a different type of organised extracurricular activity. Respondents were asked how often they take part in the following activities during their free time: (1) team sports (e.g., football, volleyball, floorball), (2) individual sports (e.g., tennis, gymnastics, karate), (3) artistic activities (e.g., playing a musical instrument, singing, dance, theatre), (4) community-based activities (e.g., scouts, YMCA, church, clubs). Respondents then rate each item on a four-point scale ranging from 1 (“I do not participate in this activity”) to 4 (“Twice a week or more”) and each activity type was treated as ordinal during the analysis.

### Data analysis

Network analysis allows us to visualize and quantify the complex web of relationships between PSMU, mental health symptoms, and substance use. **S**ince the dataset comprised a combination of continuous and categorical indicators, Mixed Graphical Models (MGMs) grounded in the Markov Random Field (MRF) framework were used to estimate the networks. While ordinal variables can theoretically be transformed (e.g., via polychoric correlations) to estimate a Gaussian Graphical Model (GGM), given our inclusion of strictly dichotomous variables (the SMDS items) alongside various ordinal measures, such transformations can lead to estimation instability or non-positive definite matrices. Therefore, MGMs were deemed more appropriate as they explicitly accommodate the distinct distributions of mixed data types. In these graphical models, nodes represent observed variables (e.g., a specific symptom or behavior), and edges (the lines connecting them) indicate conditional associations between pairs of variables after adjusting for all other variables in the network [42]. The choice of MRF-based modelling was motivated by its ability to (1) estimate partial associations while statistically controlling for the entire network [43], (2) identify reciprocal dependencies such as feedback mechanisms between connected variables [44], and (3) enable the exploration of complex, item-level dependencies and the detection of key nodes, as well as the examination of network reconfiguration following the inclusion of new nodes [42].

MGM estimation followed a generalized node-wise regression, where each variable is predicted by all others [45]. Edges were retained only if present in both complementary regressions involving the same pair of nodes, following the “AND” rule. To reduce spurious associations, Least Absolute Shrinkage and Selection Operator (LASSO) regularisation with the Extended Bayesian Information Criterion (EBIC) was used. The tuning parameter γ was fixed at 0.5 to prioritise parsimony and mitigate the inclusion of false-positive connections while maintaining theoretically meaningful edges [46, 47]. Afterwards, a sensitivity analysis was conducted to assess the replication of our findings under more conservative conditions setting γ at 0.75. Networks were visualised using the Fruchterman–Reingold spring layout, which distributes nodes in a way that balances attraction and repulsion forces, improving interpretability [48]. Node positions were subsequently fixed to allow direct visual comparison between networks estimated including different extracurricular activities.

Predictability of each node was quantified by *R²* for continuous variables and by classification accuracy for categorical ones, expressing how much variance in a node could be accounted for by its neighbours [49]. Expected influence was used as an indicator of a node’s overall strength and its relative importance within the network. To ensure robustness, the reliability of both edge weights and centrality metrics was examined using the bootnet package [42]. Non-parametric bootstrapping with 2,500 resamples was applied to assess edge-weight stability, while the case-dropping bootstrap, also based on 2,500 samples, was used to estimate the stability of centrality indices [42]. Tests for missing data pattern showed no evidence of non-random missingness according to Little’s MCAR test. On this basis, records with missing entries were excluded from the analyses using a listwise deletion.

The analytical strategy proceeded in two main steps:

In the first step, an overall network was estimated using all variables (PSMU, mental health, substance use, and demographics) to establish baseline associations. In the subsequent step, four additional networks were estimated, adding an extracurricular activity type (artistic activities, community-based activities, individual sports, and team sports) to the full sample baseline model to examine the unique role of each extracurricular activity. This allows us to visualise and quantify the direct conditional associations between that specific activity and all other PSMU, mental health, and substance use variables, while accounting for all other relationships in the network. It is important to note that while the full sample is representative of Czech adolescents, the networks that include a specific activity node represent the full sample and estimate the associations related to that activity. They do not represent networks derived only from the sub-sample of adolescents who participate in that activity.

## Results

### Descriptive statistics

Table 1 presents the item-level distribution of the nine Social Media Disorder Scale items and the single-item preference for online emotional expression, reported separately for boys and girls. Table 2 then summarises participation in the four extracurricular activity types by gender.

**Table 1:** Descriptive statistics of problematic social media use scale items and preference for online emotional expression among Czech adolescents in the 2022 Health Behaviour in School-aged Children study

Social Media Disorder Scale (SMDS) items	Boys (*N* = 7395)	Girls (*N* = 7193)
SM1		
No	5251 (71.0%)	4877 (67.8%)
Yes	1347 (18.2%)	1667 (23.2%)
Missing	797 (10.8%)	649 (9.0%)
SM2		
No	5493 (74.3%)	5291 (73.6%)
Yes	1083 (14.6%)	1251 (17.4%)
Missing	819 (11.1%)	651 (9.1%)
SM3		
No	5433 (73.5%)	5096 (70.8%)
Yes	1130 (15.3%)	1436 (20.0%)
Missing	832 (11.3%)	661 (9.2%)
SM4		
No	4673 (63.2%)	3787 (52.6%)
Yes	1864 (25.2%)	2717 (37.8%)
Missing	858 (11.6%)	689 (9.6%)
SM5		
No	5449 (73.7%)	5172 (71.9%)
Yes	1092 (14.8%)	1341 (18.6%)
Missing	854 (11.5%)	680 (9.5%)
SM6		
No	5495 (74.3%)	5284 (73.5%)
Yes	1024 (13.8%)	1192 (16.6%)
Missing	876 (11.8%)	717 (10.0%)
SM7		
No	5656 (76.5%)	5418 (75.3%)
Yes	888 (12.0%)	1082 (15.0%)
Missing	851 (11.5%)	693 (9.6%)
SM8		
No	4240 (57.3%)	3000 (41.7%)
Yes	2279 (30.8%)	3503 (48.7%)
Missing	876 (11.8%)	690 (9.6%)
SM9		
No	5699 (77.1%)	5393 (75.0%)
Yes	836 (11.3%)	1112 (15.5%)
Missing	860 (11.6%)	688 (9.6%)
SMP		
Strongly disagree	3055 (41.3%)	2419 (33.6%)
Disagree	1186 (16.0%)	1207 (16.8%)
Neither agree nor disagree	1274 (17.2%)	1527 (21.2%)
Agree	712 (9.6%)	854 (11.9%)
Strongly agree	445 (6.0%)	620 (8.6%)
Missing	723 (9.8%)	566 (7.9%)

Values are presented as n (%); percentages are column percentages calculated within each gender group and include missing responses. N = number of respondents. SMDS = Social Media Disorder Scale; items SM1–SM9 were answered yes/no with reference to the past 12 months. SM1 = can’t think of anything else but using social media; SM2 = felt dissatisfied because wanted to use social media; SM3 = felt bad when could not use social media; SM4 = failed to spend less time on social media; SM5 = lost interest in other activities because of social media use; SM6 = had arguments with others because of social media use; SM7 = lied to others about the amount of time spent on social media; SM8 = used social media to escape negative feelings; SM9 = had serious conflict with family because of social media use. SMP = preference to share one’s own feelings through social media rather than face-to-face, rated on a five-point scale from “strongly disagree” to “strongly agree.”

**Table 2:** Descriptive statistics of extracurricular activity participation by gender among Czech adolescents in the 2022 Health Behaviour in School-aged Children study with socio-demographic comparison

Variable	Boys (*n* = 7395)	Girls (*n* = 7193)	*p*
Individual sports, median [IQR]	1.00 [1.00–3.00]	1.00 [1.00–3.00]	< 0.001
Team sports, median [IQR]	3.00 [1.00–4.00]	1.00 [1.00–3.00]	< 0.001
Community-based activities, median [IQR]	1.00 [1.00–1.67]	1.00 [1.00–1.67]	
Artistic activities, median [IQR]	1.00 [1.00–2.00]	2.00 [1.00–4.00]	< 0.001

Values are medians with interquartile ranges [IQR]. Each activity was self-reported on a four-point frequency scale ranging from 1 (“I do not participate in this activity”) to 4 (“twice a week or more”), with higher values indicating more frequent participation. p = p-value for the gender difference, tested with the Mann-Whitney U test using the psychtoolbox package in R [50]: Yuen’s test and Mann-Whitney U test. n = number of respondents

### Network analysis

**Fig. 1:**
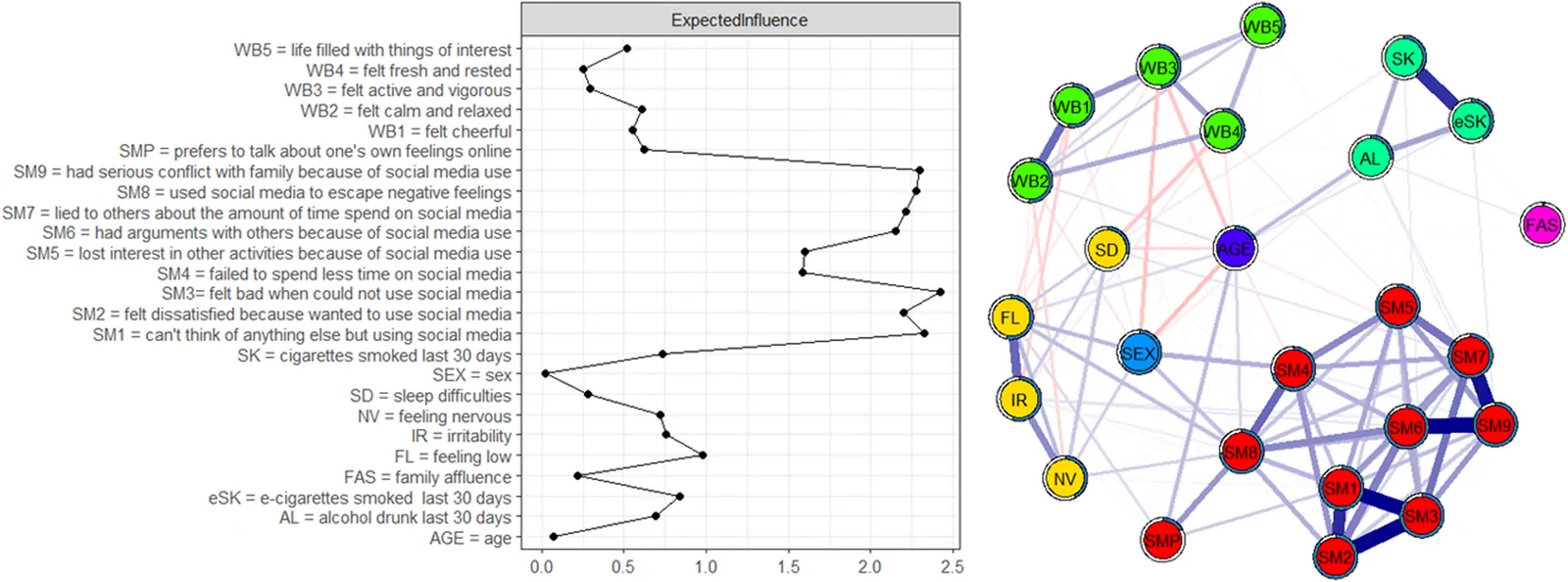
Estimated network of problematic social media use, mental-health, well-being, and substance-use indicators, with expected influence centrality, among Czech adolescents in the 2022 Health Behaviour in School-aged Children study. Note. The network was estimated on the full sample (*N* = 14,588) of Czech adolescents and adjusted for age, gender, and family affluence. Nodes represent observed variables; edges represent regularised partial associations (conditional dependencies) between pairs of variables, controlling for all others. Thicker edges indicate stronger associations; blue edges indicate positive associations and red edges negative associations. The blue-shaded portion of the ring around each node indicates node predictability (variance explained by neighbouring nodes; R² for continuous variables and classification accuracy for categorical variables). The right-hand panel shows expected influence centrality, a structural index of how strongly and in which direction each node is connected within the network (higher values indicate stronger overall connectivity); it does not denote a causal effect. SM1–SM9 = Social Media Disorder Scale (SMDS) items; SMP = preference for online emotional expression; WB1–WB5 = World Health Organization–Five Well-Being Index (WHO-5) items; SD = sleeping difficulty; FL = feeling low; NV = nervousness; IR = irritability; AL = drinking alcohol; SK = smoking tobacco; eSK = smoking e-cigarettes; FAS = Family Affluence Scale

In the first step, an overall full sample network adjusted for age, gender, and FAS was estimated and inspected (Fig. 1). Complete node predictability results are discussed in Supplementary Material 1. All correlation stability coefficients remained confidently above 0.25 threshold. The network revealed several positive associations (i.e., non-zero edges) between PSMU items and mental health complaints. The strongest links were observed between feeling low and SM8 (“using social media to escape negative feelings”; w = 0.15) as well as SMP (“preference to talk about feeling online”; w = 0.08). Associations were also found between nervousness and SM8 (w = 0.11) and sleeping difficulty and SM7 (“lying about the amount of time spent on social media”; w = 0.06). These edges remained stable even under more conservative regularisation. Weaker links were observed between irritability and SM8 (w = 0.08), SM6 (“having arguments with others because of social media use”; w = 0.06), and SM9 (“having conflicts with family because of social media use”; w = 0.05). All edges, except between SM9 and irritability, remained in the network under stricter conditions.

Several relatively weaker edges were observed between PSMU and substance use items. Specifically between cigarette smoking and SM3 (“feeling bad when not using social media”; w = 0.05), electronic cigarette smoking and SM9 (w = 0.05), and drinking alcohol and SM7 (w = 0.04), as well as SM9 (w = 0.04). All edges, except between drinking alcohol and SM7, were still observed with stronger regularisation. However, only one direct edge was found between well-being and PSMU even under more conservative conditions, specifically between WB3 (“feeling active and vigorous”) and SM5 (“losing interest in hobbies or other activities due to social media use”; w = 0.04). See Supplementary Material 2 for more comprehensive sensitivity analysis findings. For a complete correlation matrix, estimated edge weights with confidence intervals and edge thickness visualisation of the overall full sample network see Supplementary Material 3.

See Supplementary Material 4, for comprehensive bootstrapping results, including network centrality and edge difference tests, edge-weight accuracy metrics, and stability analysis. The nodes with the highest expected influence centrality were SM1, SM3, and SM9, whereas the least central node was WB4 (excluding age, gender, and FAS).

**Fig. 2:**
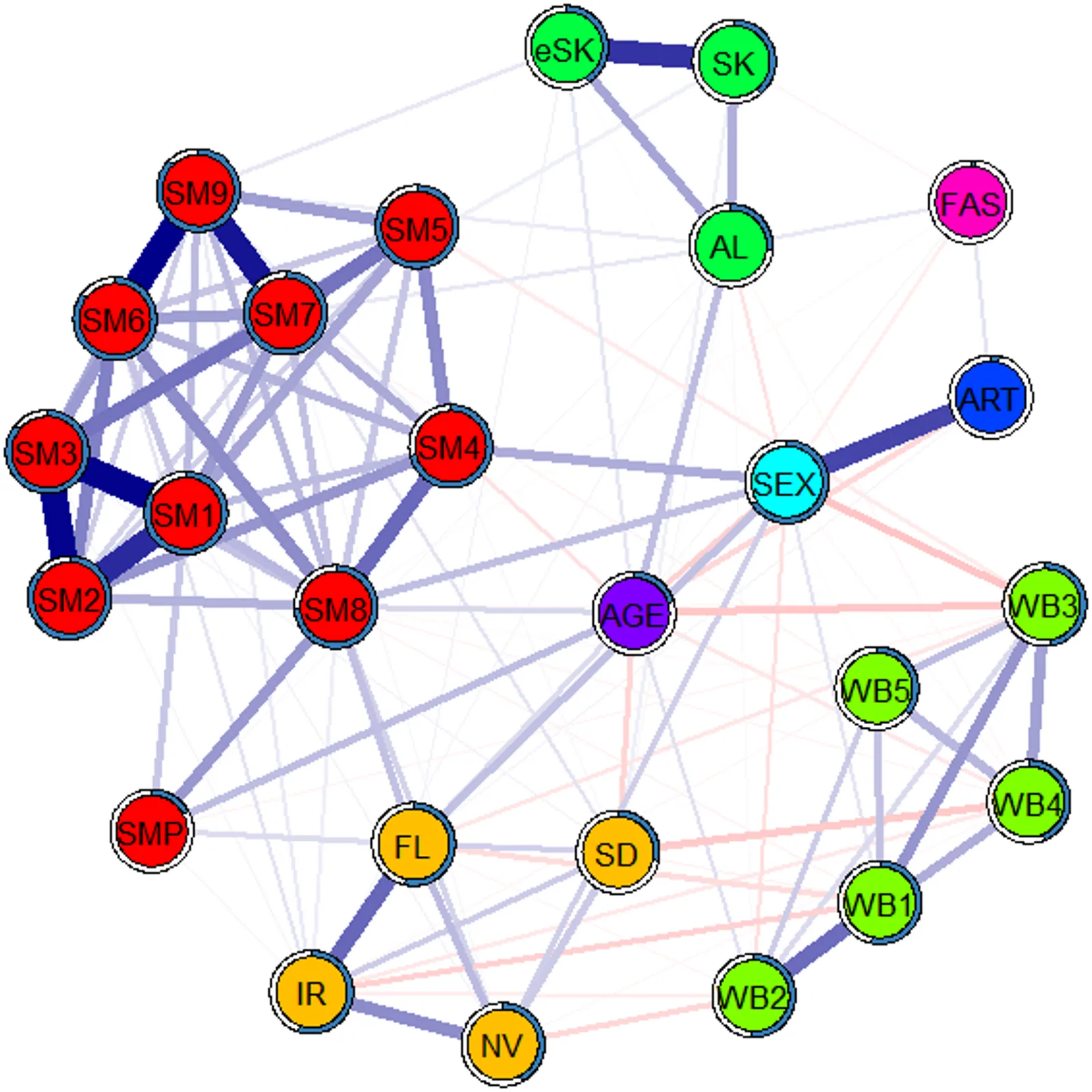
Estimated network of problematic social media use, mental-health, well-being, and substance-use including artistic activities, among Czech adolescents in the 2022 Health Behaviour in School-aged Children study. *Note.* The network was estimated on the full sample (*N* = 14,588) of Czech adolescents and adjusted for age, gender, and family affluence. Nodes represent observed variables; edges represent regularised partial associations (conditional dependencies) between pairs of variables, controlling for all others. Thicker edges indicate stronger associations; blue edges indicate positive associations and red edges negative associations. The blue-shaded portion of the ring around each node indicates node predictability (variance explained by neighbouring nodes; R² for continuous variables and classification accuracy for categorical variables). SM1–SM9 = Social Media Disorder Scale (SMDS) items; SMP = preference for online emotional expression; WB1–WB5 = World Health Organization–Five Well-Being Index (WHO-5) items; SD = sleeping difficulty; FL = feeling low; NV = nervousness; IR = irritability; AL = drinking alcohol; SK = smoking tobacco; eSK = smoking e-cigarettes; FAS = Family Affluence Scale; ART = artistic activities

Subsequently, a full sample network that additionally included artistic activities (ART) was estimated and assessed (Fig. 2). However, no differences in the network’s edge structure were observed compared to the baseline network. Additionally, no non-zero edges were present between artistic activities and PSMU nodes. All correlation stability coefficients stayed above 0.25 threshold. For a complete correlation matrix, estimated edge weights with confidence intervals and visual edge thickness visualisation see Supplementary Material 5. See Supplementary Material 6, for comprehensive bootstrapping results, including network centrality and edge difference tests, edge-weight accuracy metrics, and stability analysis.

**Fig. 3:**
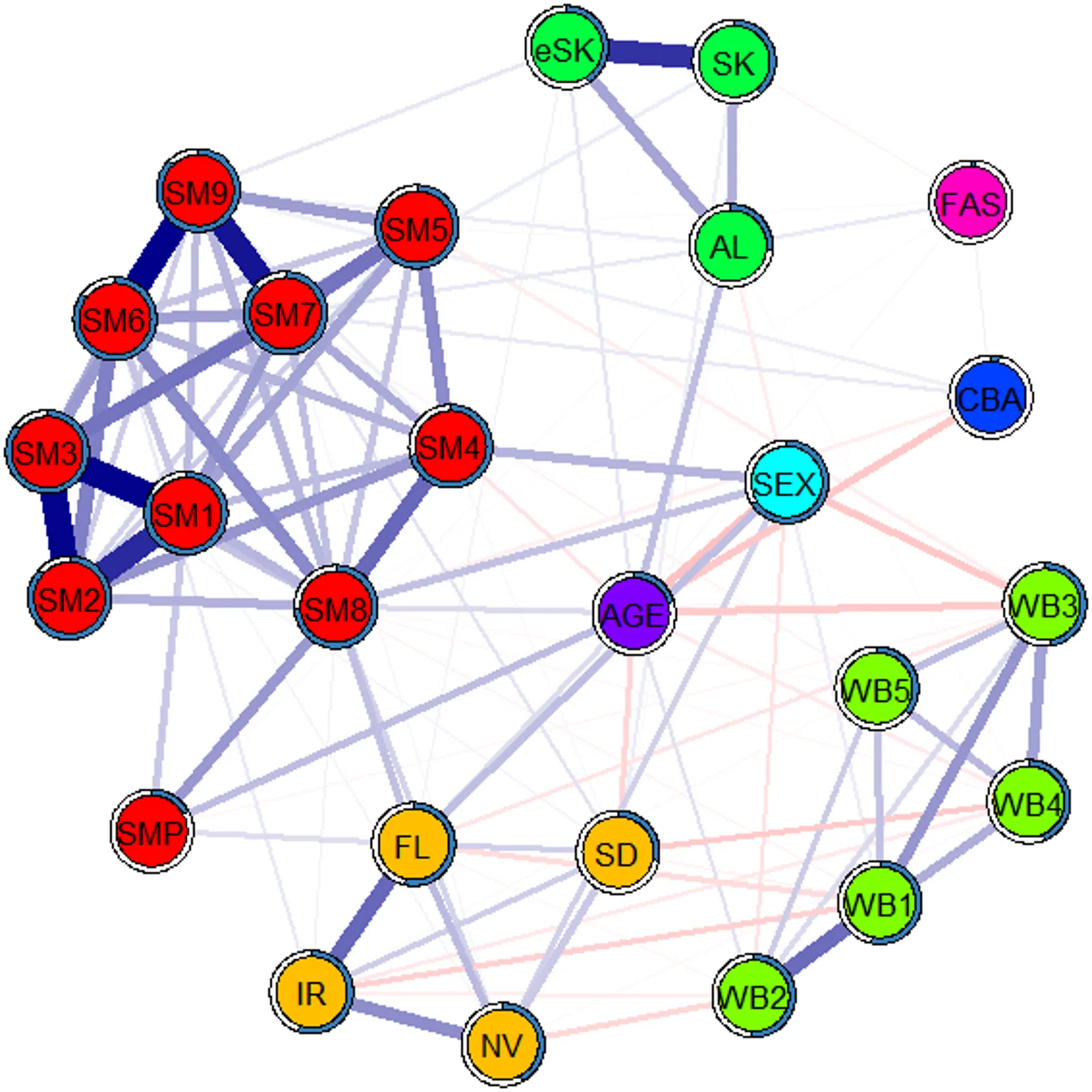
Estimated network of problematic social media use, mental-health, well-being, and substance-use including community-based activities, among Czech adolescents in the 2022 Health Behaviour in School-aged Children study. Note. The network was estimated on the full sample (*N* = 14,588) of Czech adolescents and adjusted for age, gender, and family affluence. Nodes represent observed variables; edges represent regularised partial associations (conditional dependencies) between pairs of variables, controlling for all others. Thicker edges indicate stronger associations; blue edges indicate positive associations and red edges negative associations. The blue-shaded portion of the ring around each node indicates node predictability (variance explained by neighbouring nodes; R² for continuous variables and classification accuracy for categorical variables). SM1–SM9 = Social Media Disorder Scale (SMDS) items; SMP = preference for online emotional expression; WB1–WB5 = World Health Organization–Five Well-Being Index (WHO-5) items; SD = sleeping difficulty; FL = feeling low; NV = nervousness; IR = irritability; AL = drinking alcohol; SK = smoking tobacco; eSK = smoking e-cigarettes; FAS = Family Affluence Scale; CBA = community-based activities

Afterwards, a full-sample network that included community-based activities (CBA) was estimated and inspected (Fig 3). Notable differences were observed in the associations between irritability and PSMU. Specifically, the edge between irritability and SM9 (“having conflicts with family because of social media use”) was not found in this network, and the edge weight between irritability and SM8 (“using social media to escape negative feelings”) was smaller compared to the overall network (w = 0.08 → 0.03). Non-zero positive edges were also observed between community-based activities and PSMU, specifically SM7 (“lying about the amount of time spent on social media”; w = 0.05) and SM9 (w = 0.04). All correlation stability coefficients were above 0.25 threshold. For a complete correlation matrix, estimated edge weights with confidence intervals and visual edge thickness visualisation see Supplementary Material 7. See Supplementary Material 8, for comprehensive bootstrapping results, including network centrality and edge difference tests, edge-weight accuracy metrics, and stability analysis.

**Fig. 4:**
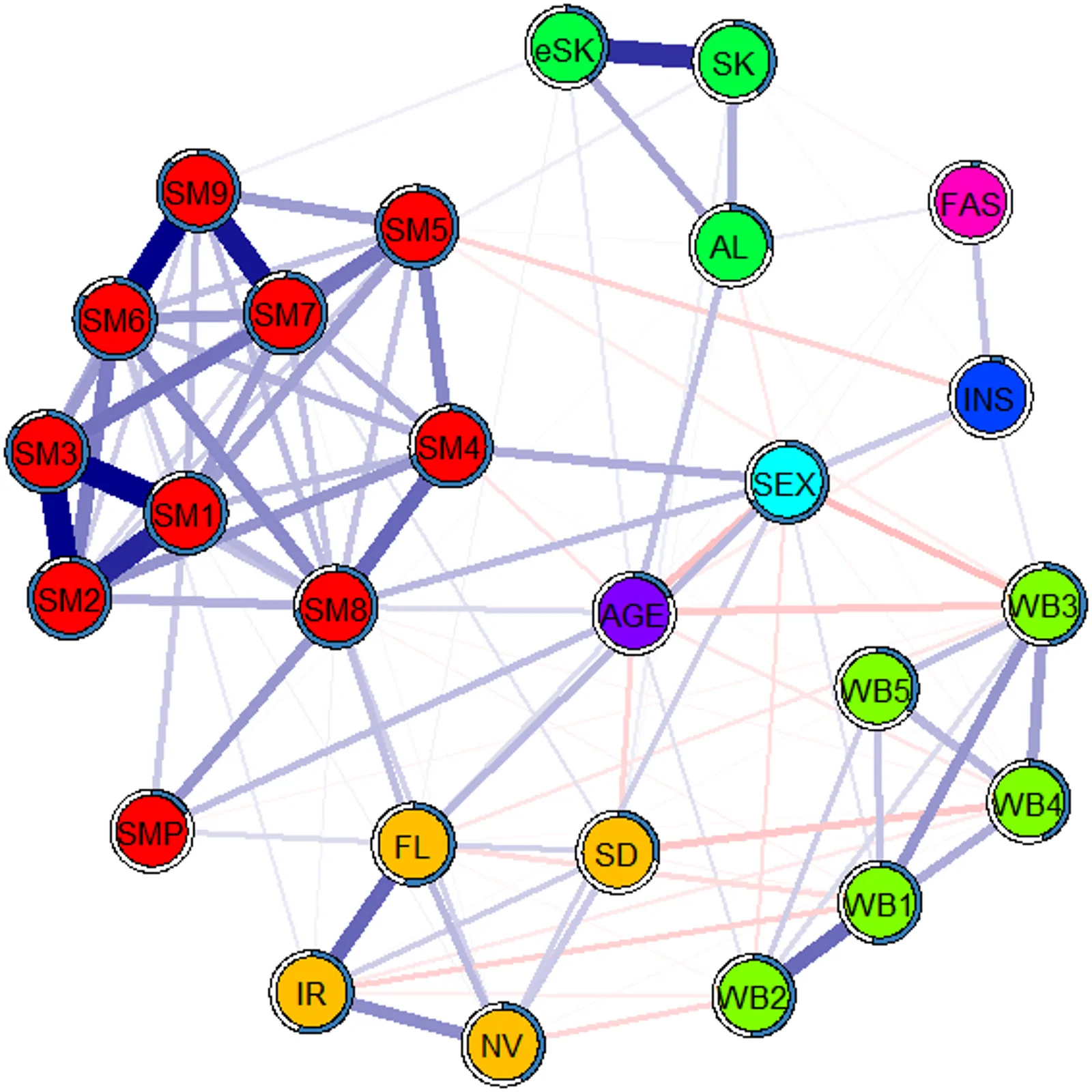
Estimated network of problematic social media use, mental-health, well-being, and substance-use including individual sports, among Czech adolescents in the 2022 Health Behaviour in School-aged Children study. Note. The network was estimated on the full sample (*N* = 14,588) of Czech adolescents and adjusted for age, gender, and family affluence. Nodes represent observed variables; edges represent regularised partial associations (conditional dependencies) between pairs of variables, controlling for all others. Thicker edges indicate stronger associations; blue edges indicate positive associations and red edges negative associations. The blue-shaded portion of the ring around each node indicates node predictability (variance explained by neighbouring nodes; R² for continuous variables and classification accuracy for categorical variables). SM1–SM9 = Social Media Disorder Scale (SMDS) items; SMP = preference for online emotional expression; WB1–WB5 = World Health Organization–Five Well-Being Index (WHO-5) items; SD = sleeping difficulty; FL = feeling low; NV = nervousness; IR = irritability; AL = drinking alcohol; SK = smoking tobacco; eSK = smoking e-cigarettes; FAS = Family Affluence Scale; INS = individual sports

Next, a full-sample network that included individual sports (INS) was estimated and inspected (Fig. 4). Compared to the baseline network, differences in edge structure were observed across two clusters. First, similarly to the CBA network, the edge between irritability and SM9 (“having conflicts with family because of social media use”) was absent, and the edge weight between irritability and SM8 (“using social media to escape negative feelings”) was smaller (w = 0.08 → 0.03). Second, the edges between drinking alcohol and both previously connected PSMU items (SM7 and SM9) were also absent in this network. Additionally, a negative edge was observed between INS and SM5 (“losing interest in hobbies or other activities due to social media use”; w = − 0.10). All correlation stability coefficients remained above 0.25 threshold. For a complete correlation matrix, estimated edge weights with confidence intervals and visual edge thickness visualisation see Supplementary Material 9. See Supplementary Material 10, for comprehensive bootstrapping results, including network centrality and edge difference tests, edge-weight accuracy metrics, and stability analysis.

**Fig. 5:**
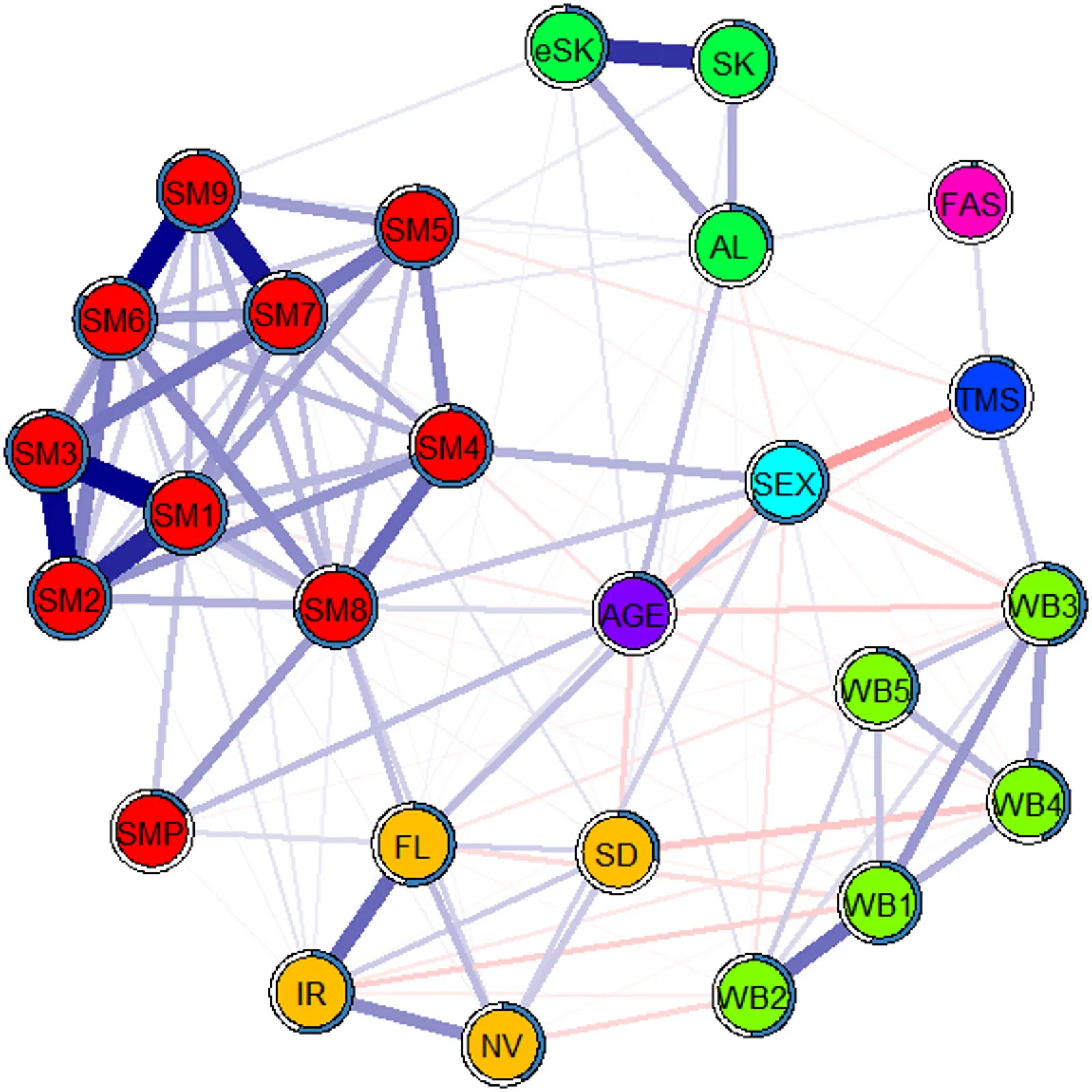
Estimated network of problematic social media use, mental-health, well-being, and substance-use including team sports, among Czech adolescents in the 2022 Health Behaviour in School-aged Children study. Note. The network was estimated on the full sample (*N* = 14,588) of Czech adolescents and adjusted for age, gender, and family affluence. Nodes represent observed variables; edges represent regularised partial associations (conditional dependencies) between pairs of variables, controlling for all others. Thicker edges indicate stronger associations; blue edges indicate positive associations and red edges negative associations. The blue-shaded portion of the ring around each node indicates node predictability (variance explained by neighbouring nodes; R² for continuous variables and classification accuracy for categorical variables). SM1–SM9 = Social Media Disorder Scale (SMDS) items; SMP = preference for online emotional expression; WB1–WB5 = World Health Organization–Five Well-Being Index (WHO-5) items; SD = sleeping difficulty; FL = feeling low; NV = nervousness; IR = irritability; AL = drinking alcohol; SK = smoking tobacco; eSK = smoking e-cigarettes; FAS = Family Affluence Scale; TMS = team sports

Lastly, a full-sample network that included team sports (TMS) was estimated and inspected (Fig. 5). A negative edge was observed between TMS and SM5 (“losing interest in hobbies or other activities due to social media use”; w = − 0.06). However, no other differences in the network’s edge structure were observed compared to the baseline network. All correlation stability coefficients remained well above 0.25 threshold. For a complete correlation matrix, estimated edge weights with confidence intervals and visual edge thickness visualisation see Supplementary Material 11. See Supplementary Material 12, for comprehensive bootstrapping results, including network centrality and edge difference tests, edge-weight accuracy metrics, and stability analysis.

#### Summary of findings

Table 3 summarises changes in network edge structure following the inclusion of different types of extracurricular activities.

**Table 3:** Summary of changes in network edge structure after including each extracurricular activity type, relative to the baseline network, among Czech adolescents in the 2022 Health Behaviour in School-aged Children study

Network model (Figure)	Observed changes compared to the baseline network model (Fig. 1)
ART (Fig. 2)	No edge-structure differences observed
CBA (Fig. 3)	(-) Irritability–SM9 (*) Irritability–SM8 (0.08 → 0.03) (+) CBA–SM7 (0.05), CBA–SM9 (0.04)
INS (Fig. 4)	(-) Irritability–SM9, Alcohol–SM7, Alcohol–SM9 (*) Irritability–SM8 (0.08 → 0.03) (+) INS–SM5 (− 0.10)
TMS (Fig. 5)	(+) TMS–SM5 (− 0.06)

Changes are described relative to the baseline full-sample network (Fig. 1). (−) = an edge present in the baseline network was no longer observed; (*) = an edge decreased in strength (values show the baseline → model edge weight); (+) = a new edge was observed (the value in parentheses is its regularised partial-association weight, including sign). ART = artistic activities; CBA = community-based activities; INS = individual sports; TMS = team sports; IR = irritability; AL = drinking alcohol; SM5 = lost interest in other activities because of social media use; SM7 = lied to others about the amount of time spent on social media; SM8 = used social media to escape negative feelings; SM9 = had serious conflict with family because of social media use

## Discussion

The main objective of the present study was to examine whether the association between PSMU, mental health symptoms and substance use differed in adolescents participating in different types of extracurricular activities (individual sports, team sports, art, and community-based activities). The results revealed that when including individual sports in the network model, the association between alcohol use and PSMU became non-significant. Furthermore, after including either individual sports or community-based extracurricular activities in the model, the correlation between irritability and family conflicts due to PSMU was non-significant. Also, the association between irritability and social media escapism was weaker in the models including individual sports or community-based extracurricular activities. Nonetheless, only sports activities were negatively associated with losing interest in extracurricular activities due to PSMU. In contrast, participation in community-based activities was positively associated with lying about the time spent on social media and family conflicts due to PSMU. Participation in artistic activities and team sports were not associated by structural changes in the network.

Initially, PSMU showed a positive association with alcohol use. Crucially, however, in our network analysis that included participation in individual sports, this association was no longer observed. Consistent with our initial observations, numerous studies have reported that youths who engage in PSMU are at elevated risk for substance use [51–53]. Psychosocially, online platforms expose adolescents to peer norms and marketing that can normalize drinking [54, 55]. Indeed, experimental research has shown that greater exposure to alcohol content on social media is linked to stronger alcohol abuse in young adolescents [56, 57]. The idea that constructive extracurricular activities can mitigate adolescent risk behaviors is supported by past research, though prior work often did not isolate individual vs. team contexts [58, 59]. However, the degree of protection appears to vary by activity type [60, 61]. Earlier research tended to lump all sports together and often found mixed results. While athletic involvement is linked with positive outcomes like higher physical activity and fitness, it has also been associated with greater alcohol use in youth cohorts [62]. Our data help reconcile this apparent paradox by implicating the type of sport as a key factor. Recent studies focusing on sport subtypes have indeed noted that the pro-alcohol attitudes are largely driven by team sports contexts [61–63]. Murray and colleagues (2021) [63] showed that high school team-sport participants, especially those in traditionally high-contact, male-typed sports like football, reported significantly more frequent drinking and binge drinking. By contrast, some studies point out that athletes in individual sports (e.g. athletics, swimming, tennis) generally exhibit lower rates of alcohol misuse than team-sport athletes. For example [61], found that college students competing in individual sports were far less likely to be hazardous drinkers and drank substantially less on average than those in team sports. The study also reported an intriguing interaction; among individual-sport athletes, a stronger athletic identity was associated with decreased alcohol consumption, whereas in team-sport athletes a strong sports identity actually predicted higher alcohol use [61]. This implies that the values and norms internalized in individual sports (e.g. personal health, self-discipline, introspection) diverge from those in some team sports (where camaraderie may involve partying), leading to different outcomes.

Furthermore, the results revealed that in the models including the participation in individual sports or community-based activities, the key associations in the PSMU–mental health network were weaker. Specifically, when including involvement in either individual sports or community activities, a previously significant link between irritability and family conflicts due to PSMU was no longer observed, and the connection between irritability and using social media for escapism was substantially smaller than in the original model. Indeed, previous work has shown that frequent social media users report greater irritability even after accounting for baseline anxiety or depression [20, 64]. Additionally, past studies concluded that youth from conflict-ridden families are at increased risk of PSMU [65]. Adolescents often turn to online activities as an escape or emotion-regulation strategy in the face of family struggles [66, 67]. Prior research broadly agrees that youth engagement in extracurriculars is associated with better mental health and lower PSMU [28, 68]. However, our results refine this general principle by indicating that the type of activity matters. Only individual sports and community-based activities were associated with absence of the edge between irritability and PSMU, whereas other activities were not. This differentiation finds some precedent in the literature. Sports participation in general is often associated with positive youth outcomes, but team-based and individual sports may confer different psychosocial benefits [69–71]. Hoffmann and colleagues (2022) [70] found that team sports participation correlated with fewer mental health difficulties, including lower anxiety and depressive symptoms, compared to non-sport participation. Counterintuitively to our findings, youth who participated exclusively in individual sports showed greater emotional and behavioral difficulties than non-athletes on average [70, 71], potentially because factors like performance pressure or lack of team support might stress some individual sport athletes. Individual sports participation may capture underlying vulnerability linked to both PSMU and mental health problems, and that is why the association between PSMU and mental health was no longer observed. Also, while previous studies have shown that adolescents participating in individual sports report poorer mental health and more problematic behaviours, findings on specific symptoms show a more complicated pattern, with individual sports potentially contributing differently across symptom domains and accounting for the association between PSMU and only some mental health symptoms. Another possible explanation for our findings is that individual sports in particular emphasize self-discipline, personal goal-setting, emotional control, and self-regulation [72–74]. Thus, an adolescent engaged in individual sports might be better equipped to manage irritability through healthy outlets. On the front of community-based activities, previous studies showed that participation in a community youth group is associated with better self-esteem, self-efficacy, and self-worth that often lowers the likelihood of digital escapism [5, 75, 76]. These positive outcomes have been previously shown to also strengthen family bonds further negating the observed association [77–79]. Altogether, each type of extracurricular activity offers a prospective environment for fostering personal introspection and self-realization.

However, only sports activities were negatively associated with losing interest in extracurricular activities in relation to PSMU. In previous studies, physical activity and sports participation have consistently been linked to lower risk of PSMU in young people [75, 76] and our results suggest that sports involvement is associated with weaker occurrence of PSMU, specifically with losing interest in other activities because of social media use. This is consistent with some broader findings on youth activities and well-being [29, 30]. While virtually any structured activity can benefit mental health [27, 28], team sports in particular often confer distinctive advantages such as stronger social support and better emotional outcomes [69, 80]. Several plausible mechanisms may explain why sports participation (and not other extracurricular activities) was associated with a lower likelihood of losing interest in real-world activities due to PSMU. Sports involve regular and more physically intensive practice sessions, games, or training routines that inherently limit the discretionary time available for social media indulgence [81, 82]. Engagement in sport type activities frequently involves physical exhilaration, competitive excitement, and in many cases teamwork and camaraderie [80, 83]. These offline gratifications may substitute for, and thus diminish the appeal of, the virtual rewards that drive PSMU [84]. While social media immerses individuals in a virtual world, physical activities immerse them in the real world, supporting both physical and mental well-being in more tangible ways. Additionally, participation in sports often improves mood and reduces stress via well-documented physiological and psychological outcomes of exercise (e.g. endorphin release, improved sleep, stress relief) [85, 86]. This is relevant because negative mood states and mental health symptoms can drive adolescents toward maladaptive social media use as a coping mechanism [87, 88]. Moreover, more achievement based activities may further boost self-esteem, self-efficacy, and building discipline [89]. Previous research consistently showed that individuals who feel competent and positive about themselves are less likely to become over-invested in online validation or escapism [88]. Overall, the present results reinforce a growing consensus that keeping adolescents physically active and socially connected through sports can be a valuable strategy to foster real-life engagement.

In contrast, community-based activities were uniquely associated with higher endorsement of two specific PSMU-related behaviors: lying about time spent on social media and family conflicts due to PSMU. Prior research has in fact hinted that different activity domains can have distinct results on adolescent outcomes and this includes some of the negative outcomes [60–62]. Team sports participants for instance, while often faring well on academic or leadership metrics, showed higher rates of substance use than their peers [61, 62]. These patterns have been attributed to the different peer norms and cultures in each context, which might be the case even for the community-based type [30, 61]. Some emerging research even hints that youth highly engaged in more socially-based extracurriculars might use social media at equal or greater rates, albeit for different purposes. Pol and colleagues (2023) [90] observed that adolescents involved in those activities actually had higher internet and mobile use for social purposes (communication, networking, maintaining social connectedness) compared to those not in activities. This notion aligns with the “rich-get-richer” hypothesis in media research, which proposes that adolescents with strong offline social networks often extend those networks online for additional social interaction [91, 92]. Community-based programs often bring together peers from different schools or neighborhoods and involve coordinating events, group discussions, and ongoing collaboration outside of regular meetings. Adolescents in these groups are therefore likely to expand their peer network beyond their classroom and to use digital tools to maintain those connections [91, 93]. Social media and messaging platforms may become convenient for communicating with fellow group members about meet-ups, sharing information or cause-related content, and sustaining friendships formed through the activity. Crucially, this could also set the stage for conflict with parents if they perceive that the engagement starts infringing on family time. Unlike a sports practice which has a clear start and end time, community engagement can translate into more open-ended digital socializing which might result in family friction [94, 95]. Therefore, unlike sports or arts, community activities may hypothetically uniquely situate adolescents in broad social networks with potentially less structured oversight.

Contrary to expectations, neither art nor team sports participation was associated with changes in the connections between PSMU, mental health, and substance use. Arts activities have shown a complex relationship with adolescent well-being. On one hand, arts engagement is often viewed as beneficial; it can facilitate emotional expression, improve coping skills, and reduce distress [96, 97]. On the other hand, some studies indicate that teens drawn to the arts may experience more internalizing symptoms than their peers [31, 32]. The authors theorized that youth with greater negative affect might self-select into the arts as a form of coping or expression [32]. In our network analysis, this complexity may explain why art participation was not associated with discernable changes in observed edges. Even if arts offer therapeutic benefits at an individual level [97], those benefits may not translate into a weaker link between PSMU and psychological distress or risk behavior at the network level, potentially due to self-selection that cannot be addressed in the cross-sectional design. The adolescents engaging in arts might still experience the pernicious cycle of PSMU and mental health symptoms similarly to others, perhaps because many of them are managing underlying emotional vulnerabilities despite their artistic involvement [31, 32]. Those who regularly participate in more socially-based activities, are frequently engaged with social media outside of practice and games, using it for communication, entertainment, and social validation [98–100]. In other words, even those who benefit from teamwork, coaching support, and exercise could still be susceptible to the depressive and anxiety-provoking outcomes associated with PSMU. Team environments may introduce peer norms that encourage more frequent digital engagement to maintain team bonds and social connectedness even outside regular practices [98, 101], which might involve other risky behaviours [61, 62]. These nuanced patterns imply that a team sport context can simultaneously expose adolescents to some risks while protecting against others. In contrast, other activity types such as individual sports might provide unique personal benefits that are difficult to foster in alternative settings.

## Strengths and limitations

First, drawing on a large, nationally representative dataset from the Health Behaviour in School-aged Children study allowed for the detection of subtle and fine-grained associations among the study variables. Second, employing advanced analytical techniques in network analysis, namely Markov Random Fields and Mixed Graphical Models, provided a more detailed depiction of the intricate interconnections between variables, revealing how these relationships shift when including additional variables, thereby offering richer insights than those yielded by conventional statistical methods.

The current study contributes valuable findings; however, it is important to note several limitations. First, its cross-sectional approach restricts conclusions about causal relationships among the investigated variables. Future research adopting longitudinal or experimental study designs could reveal the nature of these associations, discerning directional causation, mutual interactions, or concurrent occurrences. Second, the cross-sectional design can also limit our ability to account for self-selection. Adolescents can choose specific activities based on pre-existing emotional or behavioural characteristics, which could partially or fully explain the observed associations. Third, reliance on self-reporting measurement instruments raises concerns regarding the participant responses. Respondents may unintentionally or intentionally misrepresent or inaccurately recall their social media habits or mental health status due to social desirability or recall biases, thereby affecting the reliability of the data. Fourth, the operationalization of some variables presents specific methodological constraints. Extracurricular activities were assessed using single-item measures, which lack formal validation and fail to capture the quality, context, intensity, or duration of participation. Similarly, the dichotomous (yes/no) format of the SMDS items restricts variance and may oversimplify the continuous nature of problematic behaviors, potentially reducing the sensitivity to detect subtle variations in symptom severity. Fifth, the exclusive use of Czech adolescents as the study population potentially limits the broader applicability of the findings internationally. Different cultural contexts, varying societal norms, and diverse policy contexts could significantly affect how mental health outcomes interact with problematic social media behaviours elsewhere. Sixth, while our approach of including activity types as nodes in a full-sample network provides valuable insights into their unique associations, it does not allow us to make direct claims about the experiences of adolescents who actually participate in these activities. The networks do not represent distinct sub-populations of participants, but rather show how the activity relates to other variables across the entire population. Future studies could employ multi-group network analysis to compare the network structures of adolescents who regularly participate in each activity type versus those who do not. Seventh, the use of a single item to assess preference for online emotional expression (SMP) is a limitation, as its psychometric properties are not established, therefore, the results should be interpreted with caution. Future research should employ multi-item, validated scales for this construct.

## Implications

### Implications for research

Given the cross-sectional design, these findings should guide hypothesis generation rather than inference. Future studies should use multi-wave panels and ecological momentary assessment to test whether changes in extracurricular involvement co-occur with reconfigurations in links among PSMU, mental health, and substance use. Because individual sports and community activities showed distinct associations, designs should probe selection and context by measuring frequency, competitiveness, supervision, motives, and peer norms, alongside parental monitoring. Moderated and time-varying network models could evaluate whether activity type, intensity, and timing condition bridge strength and predictability. Null findings for artistic activities and team sports warrant further sensitivity analyses. Quasi-experimental or micro-longitudinal intervention studies that alter activity exposure, combined with passive sensing and platform logs, can further assess robustness. Additionally, it is essential to replicate our findings across diverse cultural and societal contexts to reveal potential cross-national differences and ensure generalizability.

### Implications for practice

Although the observed associations were small (w = 0.03 − 0.15), they can still be practically meaningful on the population level, especially when considering the high prevalence of PSMU, mental health problems and substance use among Czech adolescents. In particular, schools and community organisations could prioritise structured individual sports, which were linked to weaker associations between alcohol use and PSMU and to fewer reports of losing interest in other activities. Since participation in individual sports and community-based activities was also related to weaker connections between irritability, escapism, and family conflict, these contexts may serve as prospective settings for developing emotion regulation and stress management skills, as well as for brief screening of irritability and social media–related family tensions. However, community-based programmes were also associated with concealment of PSMU and family conflicts, highlighting the need for staff training on recognising hidden use, promoting healthy digital habits, and encouraging open parent–adolescent communication. As artistic activities and team sports were not associated with differences in network structure, interventions in these contexts should explicitly include digital well-being components rather than assuming indirect benefits.

## Conclusions

This study highlights that the role of extracurricular activities in the network of PSMU, mental health, and substance use is far from uniform, with distinct activity types showing different patterns of association within the networks. Individual sports emerged as a salient variable, with their inclusion being associated with positive changes between alcohol use, irritability, and PSMU-related symptoms. Community-based activities, by contrast, exhibited a more nuanced role, being associated with weaker irritability-related conflicts about PSMU and stronger associations with PSMU-related deception and conflicts. Artistic activities and team sports, on the other hand, were not associated with structural changes in the network. Collectively, these findings challenge the tendency to treat extracurricular engagement as a uniform construct and underscore the value of differentiating between activity types.

## Supplementary Information

Supplementary Material 1.

Supplementary Material 2.

Supplementary Material 3.

Supplementary Material 4.

Supplementary Material 5.

Supplementary Material 6.

Supplementary Material 7.

Supplementary Material 8.

Supplementary Material 9.

Supplementary Material 10.

Supplementary Material 11.

Supplementary Material 12.

## Data Availability

Data, programming scripts, and additional resources linked to this research can be accessed through the following Open Science Framework (OSF) link: https://osf.io/9j4qr
