# Supplementary material for: Problematic social media use, mental health, and substance use in Czech adolescents: the comparative role of extracurricular activities — findings from the 2022 HBSC study
Source: Arch Public Health. 2026 Jun 16;84:192. doi: 10.1186/s13690-026-01993-1 (PMC13508371; doi:10.1186/s13690-026-01993-1)
Supplement: Supplementary file 3 [file 13690_2026_1993_MOESM12_ESM.docx]

−1.0

−0.5

0.0

0.5

1.0

%

90

80

%

70

%

%

60

50

%

40

%

30

%

Sampled cases

Average correlation with original sample

betweenness

closeness

expectedInfluence

strength
