# Supplementary material for: Problematic social media use, mental health, and substance use in Czech adolescents: the comparative role of extracurricular activities — findings from the 2022 HBSC study
Source: Arch Public Health. 2026 Jun 16;84:192. doi: 10.1186/s13690-026-01993-1 (PMC13508371; doi:10.1186/s13690-026-01993-1)
Supplement: Supplementary file 4 [file 13690_2026_1993_MOESM1_ESM.docx]

|  | **SM1** | **SM2** | **SM3** | **SM4** | **SM5** | **SM6** | **SM7** | **SM8** | **SM9** | **SMP** | **FL** | **IR** | **NV** | **SD** | **WB1** | **WB2** | **WB3** | **WB4** | **WB5** | **SK** | **eSK** | **AL** | **SEX** | **TMS** | **AGE** | **FAS** |
| --- | --- | --- | --- | --- | --- | --- | --- | --- | --- | --- | --- | --- | --- | --- | --- | --- | --- | --- | --- | --- | --- | --- | --- | --- | --- | --- |
| SM1 | — | .637 | .660 | .383 | .446 | .476 | .517 | .433 | .495 | .272 | .220 | .266 | .254 | .227 | −.176 | −.176 | −.155 | −.197 | −.173 | .208 | .229 | .167 | .125 | −.069 | .001 | −.004 |
| SM2 |  | — | .666 | .427 | .430 | .534 | .493 | .447 | .498 | .228 | .214 | .277 | .248 | .227 | −.211 | −.201 | −.165 | −.210 | −.195 | .260 | .220 | .175 | .078 | −.084 | −.002 | −.025 |
| SM3 |  |  | — | .331 | .447 | .510 | .555 | .424 | .537 | .235 | .236 | .279 | .250 | .248 | −.182 | −.188 | −.154 | −.192 | −.184 | .291 | .256 | .193 | .124 | −.060 | .011 | .001 |
| SM4 |  |  |  | — | .388 | .385 | .395 | .436 | .344 | .164 | .200 | .214 | .198 | .192 | −.148 | −.153 | −.146 | −.172 | −.142 | .098 | .111 | .074 | .244 | −.073 | −.039 | −.028 |
| SM5 |  |  |  |  | — | .414 | .490 | .363 | .461 | .171 | .209 | .207 | .195 | .168 | −.176 | −.151 | −.179 | −.164 | −.159 | .219 | .191 | .157 | .102 | −.131 | .030 | −.041 |
| SM6 |  |  |  |  |  | — | .519 | .457 | .630 | .220 | .220 | .289 | .229 | .223 | −.166 | −.163 | −.121 | −.160 | −.142 | .250 | .238 | .184 | .073 | −.023 | .006 | −.012 |
| SM7 |  |  |  |  |  |  | — | .421 | .625 | .211 | .237 | .269 | .254 | .262 | −.209 | −.173 | −.145 | −.197 | −.201 | .225 | .217 | .196 | .104 | −.017 | −.015 | .010 |
| SM8 |  |  |  |  |  |  |  | — | .439 | .397 | .486 | .441 | .419 | .325 | −.364 | −.319 | −.339 | −.339 | −.292 | .234 | .258 | .226 | .306 | −.148 | .163 | −.049 |
| SM9 |  |  |  |  |  |  |  |  | — | .202 | .272 | .315 | .271 | .260 | −.213 | −.199 | −.151 | −.208 | −.193 | .277 | .286 | .217 | .131 | −.054 | .005 | −.006 |
| SMP |  |  |  |  |  |  |  |  |  | — | .331 | .285 | .250 | .204 | −.209 | −.177 | −.239 | −.213 | −.184 | .176 | .223 | .216 | .144 | −.102 | .251 | .004 |
| FL |  |  |  |  |  |  |  |  |  |  | — | .727 | .607 | .487 | −.521 | −.478 | −.467 | −.471 | −.365 | .327 | .326 | .255 | .382 | −.184 | .195 | −.020 |
| IR |  |  |  |  |  |  |  |  |  |  |  | — | .634 | .509 | −.537 | −.492 | −.439 | −.469 | −.386 | .288 | .299 | .235 | .297 | −.158 | .117 | −.021 |
| NV |  |  |  |  |  |  |  |  |  |  |  |  | — | .473 | −.401 | −.440 | −.357 | −.401 | −.295 | .194 | .198 | .139 | .340 | −.123 | .098 | −.012 |
| SD |  |  |  |  |  |  |  |  |  |  |  |  |  | — | −.378 | −.348 | −.316 | −.423 | −.274 | .270 | .231 | .189 | .235 | −.115 | .000 | −.013 |
| WB1 |  |  |  |  |  |  |  |  |  |  |  |  |  |  | — | .690 | .625 | .548 | .544 | −.185 | −.167 | −.154 | −.223 | .187 | −.091 | .075 |
| WB2 |  |  |  |  |  |  |  |  |  |  |  |  |  |  |  | — | .546 | .576 | .515 | −.139 | −.142 | −.100 | −.298 | .156 | −.035 | .071 |
| WB3 |  |  |  |  |  |  |  |  |  |  |  |  |  |  |  |  | — | .586 | .521 | −.213 | −.213 | −.182 | −.307 | .291 | −.212 | .072 |
| WB4 |  |  |  |  |  |  |  |  |  |  |  |  |  |  |  |  |  | — | .515 | −.237 | −.226 | −.221 | −.272 | .167 | −.152 | .037 |
| WB5 |  |  |  |  |  |  |  |  |  |  |  |  |  |  |  |  |  |  | — | −.209 | −.205 | −.142 | −.214 | .161 | −.099 | .075 |
| SK |  |  |  |  |  |  |  |  |  |  |  |  |  |  |  |  |  |  |  | — | .829 | .673 | .066 | −.074 | .376 | −.041 |
| eSK |  |  |  |  |  |  |  |  |  |  |  |  |  |  |  |  |  |  |  |  | — | .641 | .091 | −.052 | .404 | .021 |
| AL |  |  |  |  |  |  |  |  |  |  |  |  |  |  |  |  |  |  |  |  |  | — | −.016 | .003 | .413 | .086 |
| SEX |  |  |  |  |  |  |  |  |  |  |  |  |  |  |  |  |  |  |  |  |  |  | — | −.305 | −.057 | −.046 |
| TMS |  |  |  |  |  |  |  |  |  |  |  |  |  |  |  |  |  |  |  |  |  |  |  | — | −.123 | .140 |
| AGE |  |  |  |  |  |  |  |  |  |  |  |  |  |  |  |  |  |  |  |  |  |  |  |  | — | .027 |
| FAS |  |  |  |  |  |  |  |  |  |  |  |  |  |  |  |  |  |  |  |  |  |  |  |  |  | — |

**Edge Weight Matrix**

|  | **SM1** | **SM2** | **SM3** | **SM4** | **SM5** | **SM6** | **SM7** | **SM8** | **SM9** | **SMP** | **FL** | **IR** | **NV** | **SD** | **WB1** | **WB2** | **WB3** | **WB4** | **WB5** | **SK** | **eSK** | **AL** | **SEX** | **TMS** | **AGE** | **FAS** |
| --- | --- | --- | --- | --- | --- | --- | --- | --- | --- | --- | --- | --- | --- | --- | --- | --- | --- | --- | --- | --- | --- | --- | --- | --- | --- | --- |
| SM1 | — | .522 | .615 | .172 | .233 | .145 | .246 | .182 | .166 | .134 |  |  | .035 |  |  |  |  |  |  |  |  |  |  |  | .050 |  |
| SM2 |  | — | .606 | .257 | .124 | .307 | .109 | .187 | .126 |  |  |  |  |  |  |  |  |  |  |  |  |  |  |  |  |  |
| SM3 |  |  | — |  | .187 | .201 | .332 | .141 | .243 |  |  | .024 |  | .031 |  |  |  |  |  | .044 |  |  |  |  |  |  |
| SM4 |  |  |  | — | .277 | .182 | .199 | .359 | .040 |  |  |  |  | .017 |  |  |  | .013 |  |  |  |  | .179 |  | .068 |  |
| SM5 |  |  |  |  | — | .141 | .334 | .133 | .236 |  |  |  |  |  |  |  | .035 |  |  |  |  | .025 |  | .058 |  |  |
| SM6 |  |  |  |  |  | — | .229 | .279 | .612 |  |  | .063 |  |  |  |  |  |  |  |  |  |  |  |  |  |  |
| SM7 |  |  |  |  |  |  | — | .143 | .568 |  |  |  | .019 | .065 |  |  |  |  | .033 |  |  | .055 |  |  |  |  |
| SM8 |  |  |  |  |  |  |  | — | .171 | .242 | .151 | .036 | .108 |  | .033 |  | .024 | .026 | .017 |  |  |  | .139 |  | .092 |  |
| SM9 |  |  |  |  |  |  |  |  | — |  |  | .049 |  |  |  |  |  |  |  |  | .056 | .048 |  |  |  |  |
| SMP |  |  |  |  |  |  |  |  |  | — | .083 | .023 |  | .011 |  |  | .026 |  |  |  |  | .016 |  |  | .155 |  |
| FL |  |  |  |  |  |  |  |  |  |  | — | .364 | .165 | .115 | .082 | .022 | .058 |  |  |  | .032 | .013 | .159 |  | .083 |  |
| IR |  |  |  |  |  |  |  |  |  |  |  | — | .273 | .123 | .109 | .041 |  | .052 | .022 |  |  | .025 |  |  |  |  |
| NV |  |  |  |  |  |  |  |  |  |  |  |  | — | .126 |  | .093 |  | .020 |  |  |  |  | .113 |  |  |  |
| SD |  |  |  |  |  |  |  |  |  |  |  |  |  | — | .033 |  |  | .127 |  | .040 |  | .020 |  |  | .100 |  |
| WB1 |  |  |  |  |  |  |  |  |  |  |  |  |  |  | — | .352 | .248 | .017 | .146 |  |  |  | .043 |  |  |  |
| WB2 |  |  |  |  |  |  |  |  |  |  |  |  |  |  |  | — | .086 | .196 | .123 |  |  | .016 | .070 |  | .071 |  |
| WB3 |  |  |  |  |  |  |  |  |  |  |  |  |  |  |  |  | — | .223 | .157 |  |  |  | .102 | .129 | .120 |  |
| WB4 |  |  |  |  |  |  |  |  |  |  |  |  |  |  |  |  |  | — | .171 |  |  | .028 | .025 |  | .059 |  |
| WB5 |  |  |  |  |  |  |  |  |  |  |  |  |  |  |  |  |  |  | — |  | .010 |  |  |  |  |  |
| SK |  |  |  |  |  |  |  |  |  |  |  |  |  |  |  |  |  |  |  | — | .493 | .192 |  |  |  | .031 |
| eSK |  |  |  |  |  |  |  |  |  |  |  |  |  |  |  |  |  |  |  |  | — | .217 |  |  | .059 |  |
| AL |  |  |  |  |  |  |  |  |  |  |  |  |  |  |  |  |  |  |  |  |  | — | .052 | .024 | .161 | .051 |
| SEX |  |  |  |  |  |  |  |  |  |  |  |  |  |  |  |  |  |  |  |  |  |  | — | .236 | .167 |  |
| TMS |  |  |  |  |  |  |  |  |  |  |  |  |  |  |  |  |  |  |  |  |  |  |  | — | .072 | .088 |
| AGE |  |  |  |  |  |  |  |  |  |  |  |  |  |  |  |  |  |  |  |  |  |  |  |  | — | .018 |
| FAS |  |  |  |  |  |  |  |  |  |  |  |  |  |  |  |  |  |  |  |  |  |  |  |  |  | — |

**Bootstrapped 95% Confidence Intervals for Non-Zero Edge Weights**

| **Rank** | **Edge** | **Weight** | **95% CI** | **Prop. Zero** |
| --- | --- | --- | --- | --- |
| 1 | SM1–SM3 | .615 | [.542, .670] | .000 |
| 2 | SM6–SM9 | .612 | [.534, .681] | .000 |
| 3 | SM2–SM3 | .606 | [.537, .674] | .000 |
| 4 | SM7–SM9 | .568 | [.488, .645] | .000 |
| 5 | SM1–SM2 | .522 | [.449, .584] | .000 |
| 6 | SK–eSK | .493 | [.453, .533] | .000 |
| 7 | FL–IR | .364 | [.344, .384] | .000 |
| 8 | SM4–SM8 | .359 | [.310, .408] | .000 |
| 9 | WB1–WB2 | .352 | [.331, .373] | .000 |
| 10 | SM5–SM7 | .334 | [.259, .403] | .000 |
| 11 | SM3–SM7 | .332 | [.252, .411] | .000 |
| 12 | SM2–SM6 | .307 | [.228, .386] | .000 |
| 13 | SM6–SM8 | .279 | [.206, .352] | .000 |
| 14 | SM4–SM5 | .277 | [.217, .339] | .000 |
| 15 | IR–NV | .273 | [.252, .294] | .000 |
| 16 | SM2–SM4 | .257 | [.186, .321] | .000 |
| 17 | WB1–WB3 | .248 | [.229, .272] | .000 |
| 18 | SM1–SM7 | .246 | [.162, .318] | .000 |
| 19 | SM3–SM9 | .243 | [.160, .324] | .000 |
| 20 | SM8–SMP | .242 | [.211, .277] | .000 |
| 21 | SEX–TMS | −.236 | [−.270, −.209] | .000 |
| 22 | SM5–SM9 | .236 | [.159, .311] | .000 |
| 23 | SM1–SM5 | .233 | [.152, .291] | .000 |
| 24 | SM6–SM7 | .229 | [.150, .310] | .000 |
| 25 | WB3–WB4 | .223 | [.202, .244] | .000 |
| 26 | eSK–AL | .217 | [.180, .250] | .000 |
| 27 | SM3–SM6 | .201 | [.122, .280] | .000 |
| 28 | SM4–SM7 | .199 | [.133, .272] | .000 |
| 29 | WB2–WB4 | .196 | [.172, .216] | .000 |
| 30 | SK–AL | .192 | [.151, .230] | .000 |
| 31 | SM2–SM8 | .187 | [.112, .255] | .000 |
| 32 | SM3–SM5 | .187 | [.115, .261] | .000 |
| 33 | SM4–SEX | .179 | [.134, .226] | .000 |
| 34 | SM4–SM6 | .182 | [.119, .245] | .000 |
| 35 | SM1–SM8 | .182 | [.110, .239] | .000 |
| 36 | SEX–AGE | −.167 | [−.209, −.141] | .000 |
| 37 | SM8–SM9 | .171 | [.093, .255] | .000 |
| 38 | SM1–SM4 | .172 | [.104, .222] | .000 |
| 39 | WB4–WB5 | .171 | [.148, .190] | .000 |
| 40 | SM1–SM9 | .166 | [.085, .243] | .000 |
| 41 | FL–NV | .165 | [.143, .186] | .000 |
| 42 | AL–AGE | .161 | [.141, .179] | .000 |
| 43 | FL–SEX | .159 | [.125, .184] | .000 |
| 44 | WB3–WB5 | .157 | [.135, .180] | .000 |
| 45 | SMP–AGE | .155 | [.137, .175] | .000 |
| 46 | SM8–FL | .151 | [.117, .180] | .000 |
| 47 | SM7–SM8 | .143 | [.067, .219] | .003 |
| 48 | SM3–SM8 | .141 | [.071, .211] | .000 |
| 49 | WB1–WB5 | .146 | [.123, .168] | .000 |
| 50 | SM1–SM6 | .145 | [.071, .218] | .001 |
| 51 | SM5–SM6 | .141 | [.066, .214] | .001 |
| 52 | SM5–SM8 | .133 | [.068, .202] | .001 |
| 53 | SM8–SEX | .139 | [.095, .196] | .000 |
| 54 | SM1–SMP | .134 | [.088, .168] | .000 |
| 55 | WB3–TMS | .129 | [.000, .130] | .000 |
| 56 | SD–WB4 | −.127 | [−.144, −.106] | .000 |
| 57 | NV–SD | .126 | [.105, .147] | .000 |
| 58 | SM2–SM5 | .124 | [.047, .200] | .004 |
| 59 | SM2–SM9 | .125 | [.036, .210] | .008 |
| 60 | WB3–AGE | −.120 | [−.145, −.105] | .000 |
| 61 | WB2–WB5 | .123 | [.100, .145] | .000 |
| 62 | IR–SD | .122 | [.097, .142] | .000 |
| 63 | WB3–SEX | −.102 | [−.163, −.103] | .000 |
| 64 | NV–SEX | .113 | [.082, .143] | .000 |
| 65 | FL–SD | .115 | [.090, .134] | .000 |
| 66 | SM2–SM7 | .109 | [.025, .191] | .012 |
| 67 | SM8–NV | .108 | [.074, .138] | .000 |
| 68 | IR–WB1 | −.107 | [−.129, −.087] | .000 |
| 69 | SD–AGE | −.100 | [−.120, −.076] | .000 |
| 70 | NV–WB2 | −.093 | [−.112, −.074] | .000 |
| 71 | SM8–AGE | .092 | [.048, .119] | .000 |
| 72 | TMS–FAS | .088 | [.000, .088] | .000 |
| 73 | WB2–WB3 | .086 | [.061, .107] | .000 |
| 74 | FL–AGE | .083 | [.062, .103] | .000 |
| 75 | SMP–FL | .083 | [.059, .100] | .000 |
| 76 | FL–WB1 | −.082 | [−.102, −.060] | .000 |
| 77 | WB2–SEX | −.070 | [−.100, −.042] | .000 |
| 78 | TMS–AGE | .072 | [.000, .072] | .000 |
| 79 | SM4–AGE | −.068 | [−.099, −.032] | .018 |
| 80 | SM7–SD | .065 | [.000, .106] | .035 |
| 81 | SM6–IR | .063 | [.028, .103] | .014 |
| 82 | WB4–AGE | −.059 | [−.073, −.033] | .001 |
| 83 | eSK–AGE | .059 | [.037, .073] | .000 |
| 84 | SM5–TMS | −.058 | [−.097, .000] | .059 |
| 85 | SM9–eSK | .056 | [.000, .089] | .000 |
| 86 | SM7–AL | .055 | [.000, .095] | .370 |
| 87 | AL–SEX | −.052 | [−.078, .000] | .034 |
| 88 | AL–FAS | .051 | [.024, .071] | .008 |
| 89 | SM1–AGE | −.050 | [−.083, .000] | .650 |
| 90 | SM9–IR | .049 | [.000, .083] | .000 |
| 91 | SM9–AL | .048 | [.000, .088] | .450 |
| 92 | SM3–SK | .044 | [.000, .074] | .248 |
| 93 | WB1–SEX | .043 | [.000, .097] | .214 |
| 94 | SM4–SM9 | .040 | [.000, .107] | .346 |
| 95 | SD–SK | .040 | [.000, .053] | .081 |
| 96 | IR–WB2 | −.041 | [−.062, −.019] | .002 |
| 97 | SM8–IR | .036 | [.000, .066] | .000 |
| 98 | SM1–NV | .035 | [.000, .063] | .282 |
| 99 | SM5–WB3 | −.020 | [−.064, .000] | .533 |
| 100 | SM7–WB5 | −.033 | [−.075, .000] | .334 |
| 101 | FL–eSK | .032 | [.011, .045] | .020 |
| 102 | SD–WB1 | −.033 | [−.052, .000] | .031 |
| 103 | SM3–SD | .031 | [.000, .068] | .489 |
| 104 | SK–FAS | −.031 | [−.049, .000] | .196 |
| 105 | SM5–AL | .025 | [.000, .054] | .578 |
| 106 | WB4–AL | −.028 | [−.040, .000] | .174 |
| 107 | SM6–SMP | .025 | [.000, .074] | .472 |
| 108 | WB4–SEX | −.025 | [−.056, .000] | .407 |
| 109 | SM3–IR | .024 | [.000, .055] | .514 |
| 110 | AL–TMS | .024 | [.000, .042] | .284 |
| 111 | SMP–IR | .023 | [.000, .042] | .516 |
| 112 | FL–WB2 | −.022 | [−.042, .000] | .174 |
| 113 | SM8–WB3 | −.024 | [−.055, .000] | .418 |
| 114 | SM8–WB4 | −.026 | [−.053, .000] | .246 |
| 115 | IR–WB5 | −.022 | [−.038, .000] | .148 |
| 116 | NV–WB4 | −.020 | [−.040, .000] | .291 |
| 117 | SM3–SMP | .021 | [.000, .072] | .544 |
| 118 | SD–AL | .020 | [.000, .036] | .244 |
| 119 | SM8–WB1 | −.033 | [−.062, .000] | .492 |
| 120 | SM8–WB5 | −.017 | [−.047, .000] | .453 |
| 121 | NV–WB2 | −.093 | [−.112, −.074] | .000 |
| 122 | AGE–FAS | .018 | [.000, .036] | .311 |
| 123 | SM4–SD | .016 | [.000, .045] | .520 |
| 124 | WB2–AL | .016 | [.000, .035] | .000 |
| 125 | SMP–AL | .016 | [.000, .035] | .385 |
| 126 | SM7–SMP | .015 | [.000, .065] | .623 |
| 127 | SM7–TMS | .014 | [.000, .071] | .681 |
| 128 | FL–WB3 | −.058 | [−.077, −.037] | .000 |
| 129 | SMP–SD | .011 | [.000, .032] | .432 |
| 130 | FL–WB4 | −.010 | [−.033, .000] | .529 |
| 131 | FL–AL | .007 | [.000, .030] | .633 |
| 132 | SM2–SMP | .012 | [.000, .065] | .739 |
| 133 | SM5–SMP | .002 | [.000, .025] | .930 |
| 134 | WB5–eSK | −.010 | [−.026, .000] | .664 |
| 135 | SMP–WB3 | −.026 | [−.042, .000] | .194 |
| 136 | SM2–SEX | −.046 | [−.141, .000] | .510 |
| 137 | SM6–SEX | −.030 | [−.108, .000] | .552 |
| 138 | SM5–FAS | −.005 | [−.040, .000] | .838 |
| 139 | IR–AL | .025 | [.000, .038] | .444 |
