# Supplementary material for: Problematic social media use, mental health, and substance use in Czech adolescents: the comparative role of extracurricular activities — findings from the 2022 HBSC study
Source: Arch Public Health. 2026 Jun 16;84:192. doi: 10.1186/s13690-026-01993-1 (PMC13508371; doi:10.1186/s13690-026-01993-1)
Supplement: Supplementary file 5 [file 13690_2026_1993_MOESM2_ESM.docx]

|  | **SM1** | **SM2** | **SM3** | **SM4** | **SM5** | **SM6** | **SM7** | **SM8** | **SM9** | **SMP** | **FL** | **IR** | **NV** | **SD** | **WB1** | **WB2** | **WB3** | **WB4** | **WB5** | **SK** | **eSK** | **AL** | **SEX** | **INS** | **AGE** | **FAS** |
| --- | --- | --- | --- | --- | --- | --- | --- | --- | --- | --- | --- | --- | --- | --- | --- | --- | --- | --- | --- | --- | --- | --- | --- | --- | --- | --- |
| SM1 | — | .639 | .660 | .384 | .447 | .476 | .517 | .433 | .496 | .273 | .219 | .264 | .254 | .226 | −.176 | −.175 | −.154 | −.197 | −.172 | .208 | .226 | .166 | .125 | −.063 | .001 | −.004 |
| SM2 |  | — | .668 | .428 | .430 | .537 | .492 | .447 | .500 | .230 | .214 | .276 | .250 | .229 | −.214 | −.201 | −.167 | −.212 | −.197 | .264 | .223 | .178 | .079 | −.064 | .000 | −.025 |
| SM3 |  |  | — | .333 | .445 | .511 | .555 | .424 | .538 | .235 | .235 | .278 | .251 | .247 | −.182 | −.188 | −.154 | −.193 | −.184 | .290 | .255 | .192 | .124 | −.065 | .010 | .001 |
| SM4 |  |  |  | — | .390 | .385 | .396 | .434 | .344 | .164 | .198 | .213 | .197 | .190 | −.147 | −.153 | −.145 | −.172 | −.142 | .100 | .109 | .073 | .242 | −.025 | −.039 | −.027 |
| SM5 |  |  |  |  | — | .416 | .490 | .366 | .462 | .170 | .207 | .205 | .194 | .166 | −.175 | −.150 | −.179 | −.163 | −.159 | .218 | .190 | .157 | .103 | −.147 | .030 | −.042 |
| SM6 |  |  |  |  |  | — | .519 | .455 | .631 | .221 | .219 | .287 | .230 | .220 | −.164 | −.162 | −.121 | −.159 | −.138 | .252 | .236 | .184 | .070 | −.013 | .007 | −.011 |
| SM7 |  |  |  |  |  |  | — | .421 | .625 | .211 | .236 | .266 | .254 | .261 | −.209 | −.171 | −.144 | −.196 | −.199 | .226 | .216 | .196 | .105 | −.017 | −.014 | .011 |
| SM8 |  |  |  |  |  |  |  | — | .437 | .398 | .485 | .441 | .418 | .324 | −.363 | −.318 | −.338 | −.339 | −.290 | .234 | .257 | .227 | .306 | −.069 | .165 | −.048 |
| SM9 |  |  |  |  |  |  |  |  | — | .204 | .272 | .313 | .270 | .261 | −.212 | −.196 | −.151 | −.207 | −.192 | .281 | .287 | .219 | .126 | .002 | .008 | −.003 |
| SMP |  |  |  |  |  |  |  |  |  | — | .331 | .284 | .249 | .203 | −.207 | −.176 | −.238 | −.213 | −.183 | .177 | .222 | .217 | .142 | −.068 | .252 | .004 |
| FL |  |  |  |  |  |  |  |  |  |  | — | .727 | .608 | .485 | −.521 | −.478 | −.468 | −.472 | −.365 | .329 | .324 | .256 | .380 | −.031 | .197 | −.021 |
| IR |  |  |  |  |  |  |  |  |  |  |  | — | .635 | .509 | −.536 | −.491 | −.438 | −.469 | −.385 | .290 | .298 | .236 | .296 | −.047 | .119 | −.022 |
| NV |  |  |  |  |  |  |  |  |  |  |  |  | — | .472 | −.402 | −.441 | −.358 | −.401 | −.296 | .196 | .199 | .141 | .339 | −.007 | .100 | −.013 |
| SD |  |  |  |  |  |  |  |  |  |  |  |  |  | — | −.377 | −.346 | −.317 | −.423 | −.273 | .272 | .231 | .190 | .232 | −.008 | .003 | −.013 |
| WB1 |  |  |  |  |  |  |  |  |  |  |  |  |  |  | — | .690 | .625 | .548 | .543 | −.188 | −.166 | −.155 | −.219 | .055 | −.093 | .075 |
| WB2 |  |  |  |  |  |  |  |  |  |  |  |  |  |  |  | — | .546 | .575 | .515 | −.141 | −.141 | −.100 | −.295 | .036 | −.036 | .072 |
| WB3 |  |  |  |  |  |  |  |  |  |  |  |  |  |  |  |  | — | .586 | .521 | −.215 | −.212 | −.181 | −.305 | .134 | −.212 | .073 |
| WB4 |  |  |  |  |  |  |  |  |  |  |  |  |  |  |  |  |  | — | .515 | −.238 | −.226 | −.221 | −.271 | .060 | −.154 | .038 |
| WB5 |  |  |  |  |  |  |  |  |  |  |  |  |  |  |  |  |  |  | — | −.211 | −.204 | −.141 | −.212 | .101 | −.099 | .076 |
| SK |  |  |  |  |  |  |  |  |  |  |  |  |  |  |  |  |  |  |  | — | .831 | .673 | .066 | −.154 | .376 | −.040 |
| eSK |  |  |  |  |  |  |  |  |  |  |  |  |  |  |  |  |  |  |  |  | — | .641 | .088 | −.099 | .404 | .020 |
| AL |  |  |  |  |  |  |  |  |  |  |  |  |  |  |  |  |  |  |  |  |  | — | −.016 | −.033 | .413 | .086 |
| SEX |  |  |  |  |  |  |  |  |  |  |  |  |  |  |  |  |  |  |  |  |  |  | — | .108 | −.057 | −.046 |
| INS |  |  |  |  |  |  |  |  |  |  |  |  |  |  |  |  |  |  |  |  |  |  |  | — | −.104 | .201 |
| AGE |  |  |  |  |  |  |  |  |  |  |  |  |  |  |  |  |  |  |  |  |  |  |  |  | — | .026 |
| FAS |  |  |  |  |  |  |  |  |  |  |  |  |  |  |  |  |  |  |  |  |  |  |  |  |  | — |

**Edge Weight Matrix**

|  | **SM1** | **SM2** | **SM3** | **SM4** | **SM5** | **SM6** | **SM7** | **SM8** | **SM9** | **SMP** | **FL** | **IR** | **NV** | **SD** | **WB1** | **WB2** | **WB3** | **WB4** | **WB5** | **SK** | **eSK** | **AL** | **SEX** | **INS** | **AGE** | **FAS** |  |
| --- | --- | --- | --- | --- | --- | --- | --- | --- | --- | --- | --- | --- | --- | --- | --- | --- | --- | --- | --- | --- | --- | --- | --- | --- | --- | --- | --- |
| SM1 | — | .517 | .606 | .165 | .224 | .136 | .236 | .177 | .160 | .126 |  |  | .032 |  |  |  |  |  |  |  |  |  |  |  |  |  |  |
| SM2 |  | — | .605 | .252 | .119 | .311 | .098 | .184 | .122 |  |  |  |  |  |  |  |  |  |  |  |  |  |  |  |  |  |  |
| SM3 |  |  | — |  | .182 | .201 | .332 | .145 | .239 |  |  |  |  |  |  |  |  |  |  | .039 |  |  |  |  |  |  |  |
| SM4 |  |  |  | — | .286 | .183 | .196 | .358 | .027 |  |  |  |  |  |  |  |  | .013 |  |  |  |  | .194 |  | .067 |  |  |
| SM5 |  |  |  |  | — | .148 | .328 | .143 | .234 |  |  |  |  |  |  |  | .040 |  |  |  |  | .013 |  | .103 |  |  |  |
| SM6 |  |  |  |  |  | — | .231 | .278 | .605 |  |  | .050 |  |  |  |  |  |  |  |  |  |  |  |  |  |  |  |
| SM7 |  |  |  |  |  |  | — | .143 | .557 |  |  |  | .018 | .063 |  |  |  |  |  |  |  |  |  |  |  |  |  |
| SM8 |  |  |  |  |  |  |  | — | .159 | .245 | .151 | .032 | .107 |  | .032 |  | .022 | .027 |  |  |  |  | .163 |  | .089 |  |  |
| SM9 |  |  |  |  |  |  |  |  | — |  |  |  |  |  |  |  |  |  |  |  | .036 |  |  |  |  |  |  |
| SMP |  |  |  |  |  |  |  |  |  | — | .083 |  |  | .010 |  |  | .025 |  |  |  |  | .013 |  |  | .153 |  |  |
| FL |  |  |  |  |  |  |  |  |  |  | — | .364 | .166 | .112 | .081 | .023 | .060 | .014 |  |  | .029 |  | .165 |  | .082 |  |  |
| IR |  |  |  |  |  |  |  |  |  |  |  | — | .271 | .119 | .108 | .036 |  | .050 | .015 |  |  |  |  |  |  |  |  |
| NV |  |  |  |  |  |  |  |  |  |  |  |  | — | .126 |  | .094 |  | .019 |  |  |  |  | .115 |  |  |  |  |
| SD |  |  |  |  |  |  |  |  |  |  |  |  |  | — | .031 |  |  | .128 |  | .039 |  | .015 |  |  | .095 |  |  |
| WB1 |  |  |  |  |  |  |  |  |  |  |  |  |  |  | — | .353 | .251 | .017 | .143 |  |  |  | .067 |  |  |  |  |
| WB2 |  |  |  |  |  |  |  |  |  |  |  |  |  |  |  | — | .087 | .195 | .120 |  |  |  | .076 |  | .070 |  |  |
| WB3 |  |  |  |  |  |  |  |  |  |  |  |  |  |  |  |  | — | .223 | .155 |  |  |  | .155 | .058 | .124 |  |  |
| WB4 |  |  |  |  |  |  |  |  |  |  |  |  |  |  |  |  |  | — | .169 |  |  | .019 | .029 |  | .057 |  |  |
| WB5 |  |  |  |  |  |  |  |  |  |  |  |  |  |  |  |  |  |  | — |  |  |  |  |  |  |  |  |
| SK |  |  |  |  |  |  |  |  |  |  |  |  |  |  |  |  |  |  |  | — | .490 | .185 |  | .020 |  | .025 |  |
| eSK |  |  |  |  |  |  |  |  |  |  |  |  |  |  |  |  |  |  |  |  | — | .212 |  |  | .051 |  |  |
| AL |  |  |  |  |  |  |  |  |  |  |  |  |  |  |  |  |  |  |  |  |  | — | .051 |  | .153 | .047 |  |
| SEX |  |  |  |  |  |  |  |  |  |  |  |  |  |  |  |  |  |  |  |  |  |  | — | .125 | .146 | .026 |  |
| INS |  |  |  |  |  |  |  |  |  |  |  |  |  |  |  |  |  |  |  |  |  |  |  | — | .043 | .143 |  |
| AGE |  |  |  |  |  |  |  |  |  |  |  |  |  |  |  |  |  |  |  |  |  |  |  |  | — | .016 |  |
| FAS |  |  |  |  |  |  |  |  |  |  |  |  |  |  |  |  |  |  |  |  |  |  |  |  |  | — |  |

**Bootstrapped 95% Confidence Intervals for Non-Zero Edge Weights**

| **Rank** | **Edge** | **Weight** | **95% CI** | **Prop. = 0** |
| --- | --- | --- | --- | --- |
| 1 | SM6–SM9 | .612 | [.539, .688] | .000 |
| 2 | SM1–SM3 | .607 | [.544, .676] | .000 |
| 3 | SM2–SM3 | .605 | [.545, .679] | .000 |
| 4 | SM7–SM9 | .564 | [.489, .642] | .000 |
| 5 | SM1–SM2 | .515 | [.455, .587] | .000 |
| 6 | SK–eSK | .493 | [.449, .529] | .000 |
| 7 | FL–IR | .365 | [.343, .383] | .000 |
| 8 | SM4–SM8 | .360 | [.309, .407] | .000 |
| 9 | WB1–WB2 | .352 | [.331, .372] | .000 |
| 10 | SM3–SM7 | .334 | [.256, .411] | .000 |
| 11 | SM5–SM7 | .333 | [.255, .403] | .000 |
| 12 | SM2–SM6 | .306 | [.231, .392] | .000 |
| 13 | SM6–SM8 | .280 | [.222, .360] | .000 |
| 14 | SM4–SM5 | .279 | [.210, .328] | .000 |
| 15 | IR–NV | .271 | [.253, .294] | .000 |
| 16 | SM2–SM4 | .254 | [.197, .331] | .000 |
| 17 | WB1–WB3 | .251 | [.229, .272] | .000 |
| 18 | SM8–SMP | .244 | [.211, .275] | .000 |
| 19 | SM3–SM9 | .242 | [.159, .318] | .000 |
| 20 | SM1–SM7 | .241 | [.161, .312] | .000 |
| 21 | SM5–SM9 | .235 | [.152, .302] | .000 |
| 22 | SM6–SM7 | .230 | [.153, .311] | .000 |
| 23 | WB3–WB4 | .223 | [.202, .242] | .000 |
| 24 | SM1–SM5 | .222 | [.152, .289] | .000 |
| 25 | eSK–AL | .215 | [.180, .247] | .000 |
| 26 | SM3–SM6 | .201 | [.124, .282] | .000 |
| 27 | SM4–SM7 | .202 | [.128, .266] | .000 |
| 28 | WB2–WB4 | .194 | [.174, .216] | .000 |
| 29 | SK–AL | .188 | [.151, .224] | .000 |
| 30 | SM3–SM5 | .188 | [.111, .256] | .000 |
| 31 | SM2–SM8 | .184 | [.122, .261] | .000 |
| 32 | SM4–SM6 | .182 | [.130, .253] | .000 |
| 33 | SM4–SEX | .181 | [.138, .228] | .000 |
| 34 | SM1–SM8 | .174 | [.113, .241] | .000 |
| 35 | SM8–SM9 | .174 | [.092, .251] | .000 |
| 36 | WB4–WB5 | .169 | [.150, .192] | .000 |
| 37 | FL–NV | .164 | [.143, .185] | .000 |
| 38 | SM1–SM9 | .164 | [.086, .243] | .000 |
| 39 | SM1–SM4 | .163 | [.107, .220] | .000 |
| 40 | WB3–WB5 | .158 | [.135, .179] | .000 |
| 41 | SMP–AGE | .156 | [.136, .174] | .000 |
| 42 | FL–SEX | .155 | [.130, .188] | .000 |
| 43 | AL–AGE | .154 | [.137, .175] | .000 |
| 44 | SM7–SM8 | .153 | [.074, .225] | .001 |
| 45 | SM8–FL | .148 | [.117, .180] | .000 |
| 46 | WB1–WB5 | .146 | [.123, .168] | .000 |
| 47 | SM8–SEX | .145 | [.101, .202] | .000 |
| 48 | SEX–AGE | .145 | [.119, .183] | .000 |
| 49 | SM3–SM8 | .143 | [.074, .209] | .000 |
| 50 | SM5–SM6 | .139 | [.067, .211] | .000 |
| 51 | SM5–SM8 | .138 | [.068, .194] | .000 |
| 52 | SM1–SM6 | .137 | [.065, .217] | .000 |
| 53 | WB3–SEX | .129 | [.103, .163] | .000 |
| 54 | CBA–AGE | .130 | [.110, .151] | .000 |
| 55 | SM1–SMP | .129 | [.089, .168] | .000 |
| 56 | NV–SD | .126 | [.105, .145] | .000 |
| 57 | WB3–AGE | .125 | [.105, .145] | .000 |
| 58 | SM2–SM5 | .124 | [.053, .203] | .003 |
| 59 | SD–WB4 | .123 | [.106, .145] | .000 |
| 60 | WB2–WB5 | .123 | [.100, .145] | .000 |
| 61 | SM2–SM9 | .123 | [.040, .208] | .009 |
| 62 | IR–SD | .119 | [.099, .142] | .000 |
| 63 | FL–SD | .112 | [.090, .134] | .000 |
| 64 | NV–SEX | .111 | [.082, .143] | .000 |
| 65 | SM2–SM7 | .108 | [.028, .192] | .014 |
| 66 | IR–WB1 | .108 | [.088, .131] | .000 |
| 67 | SM8–NV | .106 | [.072, .136] | .000 |
| 68 | SD–AGE | .096 | [.072, .116] | .000 |
| 69 | NV–WB2 | .094 | [.074, .112] | .000 |
| 70 | WB2–WB3 | .085 | [.061, .107] | .000 |
| 71 | SM8–AGE | .083 | [.048, .119] | .000 |
| 72 | FL–AGE | .083 | [.064, .104] | .000 |
| 73 | FL–WB1 | .081 | [.059, .103] | .000 |
| 74 | SMP–FL | .080 | [.059, .100] | .000 |
| 75 | WB2–AGE | .070 | [.047, .089] | .000 |
| 76 | WB2–SEX | .068 | [.042, .100] | .000 |
| 77 | SM7–SD | .065 | [.000, .103] | .037 |
| 78 | SM4–AGE | .065 | [.033, .096] | .016 |
| 79 | FL–WB3 | .059 | [.039, .077] | .000 |
| 80 | eSK–AGE | .058 | [.037, .073] | .002 |
| 81 | SM9–eSK | .056 | [.000, .089] | .156 |
| 82 | SM7–AL | .053 | [.000, .094] | .374 |
| 83 | WB4–AGE | .053 | [.033, .073] | .001 |
| 84 | SM7–CBA | .051 | [.000, .092] | .247 |
| 85 | IR–WB4 | .050 | [.031, .072] | .001 |
| 86 | SM6–IR | .050 | [.024, .103] | .020 |
| 87 | AL–FAS | .049 | [.024, .071] | .005 |
| 88 | WB4–CBA | .049 | [.000, .035] | .496 |
| 89 | SM3–SK | .044 | [.000, .072] | .276 |
| 90 | AL–SEX | .043 | [.000, .087] | .030 |
| 91 | WB1–SEX | .042 | [.000, .097] | .214 |
| 92 | SM9–AL | .042 | [.000, .088] | .450 |
| 93 | SD–SK | .040 | [.000, .054] | .079 |
| 94 | SM4–SM9 | .039 | [.000, .104] | .360 |
| 95 | IR–WB2 | .038 | [.017, .060] | .005 |
| 96 | SM5–WB3 | .038 | [.000, .073] | .441 |
| 97 | SM8–CBA | .036 | [.000, .074] | .252 |
| 98 | SM7–WB5 | .035 | [.000, .078] | .334 |
| 99 | SM9–CBA | .035 | [.000, .082] | .515 |
| 100 | CBA–FAS | .034 | [.000, .052] | .060 |
| 101 | SD–WB1 | .034 | [.000, .053] | .026 |
| 102 | SK–FAS | .034 | [.000, .050] | .186 |
| 103 | SM8–IR | .032 | [.000, .066] | .095 |
| 104 | SM8–WB1 | .032 | [.000, .062] | .492 |
| 105 | SM1–NV | .032 | [.000, .064] | .287 |
| 106 | FL–eSK | .031 | [.012, .045] | .018 |
| 107 | SM3–SD | .031 | [.000, .068] | .492 |
| 108 | SMP–WB3 | .025 | [.000, .042] | .194 |
| 109 | SM8–WB3 | .023 | [.000, .055] | .418 |
| 110 | SM8–WB4 | .022 | [.000, .053] | .246 |
| 111 | SM7–NV | .021 | [.000, .059] | .544 |
| 112 | FL–WB2 | .021 | [.000, .042] | .166 |
| 113 | NV–WB4 | .019 | [.000, .039] | .310 |
| 114 | AGE–FAS | .018 | [.000, .036] | .396 |
| 115 | IR–WB5 | .017 | [.000, .038] | .172 |
| 116 | SM4–SD | .016 | [.000, .043] | .540 |
| 117 | SM8–WB5 | .016 | [.000, .047] | .453 |
| 118 | WB1–WB4 | .015 | [.000, .039] | .342 |
| 119 | SD–AL | .015 | [.000, .037] | .261 |
| 120 | SM5–FAS | .014 | [.000, .048] | .755 |
| 121 | SM2–SMP | .014 | [.000, .067] | .704 |
| 122 | SMP–IR | .014 | [.000, .042] | .516 |
| 123 | SMP–AL | .014 | [.000, .035] | .385 |
| 124 | WB4–AL | .014 | [.000, .040] | .174 |
| 125 | SMP–SD | .013 | [.000, .032] | .432 |
| 126 | SM5–AL | .012 | [.000, .049] | .666 |
| 127 | SM8–FAS | .012 | [.000, .044] | .656 |
| 128 | SM6–CBA | .011 | [.000, .058] | .718 |
| 129 | WB5–eSK | .010 | [.000, .026] | .664 |
| 130 | FL–WB4 | .010 | [.000, .033] | .527 |
