# Supplementary material for: Problematic social media use, mental health, and substance use in Czech adolescents: the comparative role of extracurricular activities — findings from the 2022 HBSC study
Source: Arch Public Health. 2026 Jun 16;84:192. doi: 10.1186/s13690-026-01993-1 (PMC13508371; doi:10.1186/s13690-026-01993-1)
Supplement: Supplementary file 7 [file 13690_2026_1993_MOESM4_ESM.docx]

|  | **SM1** | **SM2** | **SM3** | **SM4** | **SM5** | **SM6** | **SM7** | **SM8** | **SM9** | **SMP** | **FL** | **IR** | **NV** | **SD** | **WB1** | **WB2** | **WB3** | **WB4** | **WB5** | **SK** | **eSK** | **AL** | **SEX** | **AGE** | **FAS** |
| --- | --- | --- | --- | --- | --- | --- | --- | --- | --- | --- | --- | --- | --- | --- | --- | --- | --- | --- | --- | --- | --- | --- | --- | --- | --- |
| SM1 | — | .637 | .660 | .383 | .446 | .477 | .517 | .432 | .495 | .272 | .219 | .265 | .254 | .227 | −.177 | −.177 | −.155 | −.197 | −.173 | .209 | .229 | .167 | .126 | .001 | −.003 |
| SM2 |  | — | .666 | .427 | .430 | .536 | .493 | .446 | .499 | .228 | .214 | .278 | .249 | .227 | −.211 | −.200 | −.165 | −.209 | −.194 | .261 | .220 | .176 | .078 | −.002 | −.024 |
| SM3 |  |  | — | .331 | .447 | .511 | .555 | .424 | .537 | .234 | .236 | .280 | .250 | .247 | −.181 | −.187 | −.153 | −.192 | −.183 | .290 | .255 | .193 | .124 | .010 | .002 |
| SM4 |  |  |  | — | .388 | .386 | .395 | .436 | .345 | .164 | .200 | .214 | .198 | .192 | −.148 | −.153 | −.146 | −.172 | −.142 | .099 | .113 | .075 | .244 | −.038 | −.028 |
| SM5 |  |  |  |  | — | .414 | .490 | .364 | .461 | .171 | .209 | .208 | .195 | .168 | −.175 | −.150 | −.179 | −.164 | −.158 | .218 | .192 | .158 | .104 | .030 | −.042 |
| SM6 |  |  |  |  |  | — | .519 | .458 | .631 | .220 | .221 | .290 | .230 | .222 | −.166 | −.162 | −.121 | −.160 | −.141 | .251 | .238 | .185 | .073 | .006 | −.011 |
| SM7 |  |  |  |  |  |  | — | .421 | .624 | .210 | .237 | .268 | .254 | .260 | −.209 | −.173 | −.144 | −.198 | −.200 | .225 | .216 | .196 | .105 | −.015 | .010 |
| SM8 |  |  |  |  |  |  |  | — | .439 | .397 | .485 | .442 | .419 | .325 | −.364 | −.319 | −.339 | −.339 | −.291 | .232 | .258 | .226 | .306 | .163 | −.049 |
| SM9 |  |  |  |  |  |  |  |  | — | .204 | .272 | .316 | .272 | .261 | −.214 | −.199 | −.152 | −.209 | −.193 | .278 | .286 | .218 | .130 | .005 | −.005 |
| SMP |  |  |  |  |  |  |  |  |  | — | .331 | .285 | .250 | .205 | −.210 | −.178 | −.240 | −.214 | −.184 | .177 | .224 | .216 | .143 | .252 | .004 |
| FL |  |  |  |  |  |  |  |  |  |  | — | .727 | .607 | .486 | −.521 | −.478 | −.467 | −.471 | −.365 | .328 | .326 | .255 | .381 | .196 | −.022 |
| IR |  |  |  |  |  |  |  |  |  |  |  | — | .635 | .509 | −.537 | −.492 | −.439 | −.469 | −.386 | .288 | .299 | .236 | .296 | .118 | −.021 |
| NV |  |  |  |  |  |  |  |  |  |  |  |  | — | .473 | −.402 | −.441 | −.358 | −.401 | −.295 | .194 | .199 | .139 | .339 | .098 | −.012 |
| SD |  |  |  |  |  |  |  |  |  |  |  |  |  | — | −.379 | −.348 | −.317 | −.423 | −.274 | .270 | .232 | .190 | .234 | .001 | −.012 |
| WB1 |  |  |  |  |  |  |  |  |  |  |  |  |  |  | — | .691 | .626 | .548 | .544 | −.186 | −.167 | −.154 | −.222 | −.092 | .074 |
| WB2 |  |  |  |  |  |  |  |  |  |  |  |  |  |  |  | — | .547 | .576 | .516 | −.140 | −.143 | −.100 | −.297 | −.035 | .071 |
| WB3 |  |  |  |  |  |  |  |  |  |  |  |  |  |  |  |  | — | .586 | .521 | −.214 | −.214 | −.182 | −.306 | −.212 | .072 |
| WB4 |  |  |  |  |  |  |  |  |  |  |  |  |  |  |  |  |  | — | .516 | −.237 | −.227 | −.221 | −.271 | −.152 | .037 |
| WB5 |  |  |  |  |  |  |  |  |  |  |  |  |  |  |  |  |  |  | — | −.210 | −.206 | −.142 | −.214 | −.100 | .075 |
| SK |  |  |  |  |  |  |  |  |  |  |  |  |  |  |  |  |  |  |  | — | .829 | .674 | .066 | .376 | −.040 |
| eSK |  |  |  |  |  |  |  |  |  |  |  |  |  |  |  |  |  |  |  |  | — | .641 | .091 | .405 | .019 |
| AL |  |  |  |  |  |  |  |  |  |  |  |  |  |  |  |  |  |  |  |  |  | — | −.016 | .413 | .086 |
| SEX |  |  |  |  |  |  |  |  |  |  |  |  |  |  |  |  |  |  |  |  |  |  | — | −.057 | −.046 |
| AGE |  |  |  |  |  |  |  |  |  |  |  |  |  |  |  |  |  |  |  |  |  |  |  | — | .026 |
| FAS |  |  |  |  |  |  |  |  |  |  |  |  |  |  |  |  |  |  |  |  |  |  |  |  | — |

**Edge Weight Matrix**

|  | **SM1** | **SM2** | **SM3** | **SM4** | **SM5** | **SM6** | **SM7** | **SM8** | **SM9** | **SMP** | **FL** | **IR** | **NV** | **SD** | **WB1** | **WB2** | **WB3** | **WB4** | **WB5** | **SK** | **eSK** | **AL** | **SEX** | **AGE** | **FAS** |
| --- | --- | --- | --- | --- | --- | --- | --- | --- | --- | --- | --- | --- | --- | --- | --- | --- | --- | --- | --- | --- | --- | --- | --- | --- | --- |
| SM1 | — | .508 | .603 | .156 | .212 | .134 | .236 | .171 | .157 | .121 |  |  | .030 |  |  |  |  |  |  |  |  |  |  |  |  |
| SM2 |  | — | .604 | .252 | .120 | .305 | .104 | .184 | .125 |  |  |  |  |  |  |  |  |  |  |  |  |  |  |  |  |
| SM3 |  |  | — |  | .188 | .204 | .337 | .146 | .241 |  |  | .026 |  | .029 |  |  |  |  |  | .045 |  |  |  |  |  |
| SM4 |  |  |  | — | .275 | .183 | .203 | .361 | .040 |  |  |  |  | .016 |  |  |  | .013 |  |  |  |  | .182 | .069 |  |
| SM5 |  |  |  |  | — | .136 | .331 | .136 | .235 |  |  |  |  |  |  |  | .045 |  |  |  |  | .011 |  |  |  |
| SM6 |  |  |  |  |  | — | .232 | .282 | .611 |  |  | .064 |  |  |  |  |  |  |  |  |  |  |  |  |  |
| SM7 |  |  |  |  |  |  | — | .149 | .567 |  |  |  | .021 | .063 |  |  |  |  | .034 |  |  | .054 |  | .051 |  |
| SM8 |  |  |  |  |  |  |  | — | .171 | .243 | .151 | .037 | .108 |  | .033 |  | .024 | .026 | .018 |  |  |  | .145 | .095 |  |
| SM9 |  |  |  |  |  |  |  |  | — |  |  | .050 |  |  |  |  |  |  |  |  | .054 | .043 |  |  |  |
| SMP |  |  |  |  |  |  |  |  |  | — | .082 | .024 |  | .012 |  |  | .027 |  |  |  |  | .012 |  | .156 |  |
| FL |  |  |  |  |  |  |  |  |  |  | — | .364 | .165 | .114 | .081 | .022 | .059 | .013 |  |  | .032 |  | .160 | .085 |  |
| IR |  |  |  |  |  |  |  |  |  |  |  | — | .274 | .123 | .109 | .040 |  | .051 | .022 |  |  | .020 |  |  |  |
| NV |  |  |  |  |  |  |  |  |  |  |  |  | — | .126 |  | .094 |  | .020 |  |  |  |  | .111 |  |  |
| SD |  |  |  |  |  |  |  |  |  |  |  |  |  | — | .033 |  |  | .126 |  | .039 |  | .015 |  | .099 |  |
| WB1 |  |  |  |  |  |  |  |  |  |  |  |  |  |  | — | .353 | .252 | .017 | .146 |  |  |  | .042 |  |  |
| WB2 |  |  |  |  |  |  |  |  |  |  |  |  |  |  |  | — | .086 | .196 | .123 |  |  |  | .068 | .073 |  |
| WB3 |  |  |  |  |  |  |  |  |  |  |  |  |  |  |  |  | — | .223 | .159 |  |  |  | .135 | .133 |  |
| WB4 |  |  |  |  |  |  |  |  |  |  |  |  |  |  |  |  |  | — | .172 |  |  | .019 | .024 | .059 |  |
| WB5 |  |  |  |  |  |  |  |  |  |  |  |  |  |  |  |  |  |  | — |  | .010 |  |  |  |  |
| SK |  |  |  |  |  |  |  |  |  |  |  |  |  |  |  |  |  |  |  | — | .492 | .189 |  |  | .029 |
| eSK |  |  |  |  |  |  |  |  |  |  |  |  |  |  |  |  |  |  |  |  | — | .214 |  | .060 |  |
| AL |  |  |  |  |  |  |  |  |  |  |  |  |  |  |  |  |  |  |  |  |  | — | .043 | .154 | .046 |
| SEX |  |  |  |  |  |  |  |  |  |  |  |  |  |  |  |  |  |  |  |  |  |  | — | .148 |  |
| AGE |  |  |  |  |  |  |  |  |  |  |  |  |  |  |  |  |  |  |  |  |  |  |  | — | .008 |
| FAS |  |  |  |  |  |  |  |  |  |  |  |  |  |  |  |  |  |  |  |  |  |  |  |  | — |

**Bootstrapped 95% Confidence Intervals for Non-Zero Edge Weights**

| **Rank** | **Edge** | **Weight** | **95% CI** | **Prop. zero** |
| --- | --- | --- | --- | --- |
| 1 | SM6 – SM9 | .611 | [.542, .689] | .000 |
| 2 | SM2 – SM3 | .604 | [.548, .683] | .000 |
| 3 | SM1 – SM3 | .603 | [.547, .677] | .000 |
| 4 | SM7 – SM9 | .567 | [.486, .638] | .000 |
| 5 | SM1 – SM2 | .508 | [.454, .589] | .000 |
| 6 | SK – eSK | .492 | [.448, .528] | .000 |
| 7 | FL – IR | .364 | [.344, .383] | .000 |
| 8 | SM4 – SM8 | .361 | [.309, .407] | .000 |
| 9 | WB1 – WB2 | .353 | [.333, .372] | .000 |
| 10 | SM3 – SM7 | .337 | [.259, .411] | .000 |
| 11 | SM5 – SM7 | .331 | [.251, .397] | .000 |
| 12 | SM2 – SM6 | .305 | [.236, .394] | .000 |
| 13 | SM6 – SM8 | .282 | [.221, .362] | .000 |
| 14 | SM4 – SM5 | .275 | [.210, .331] | .000 |
| 15 | IR – NV | .274 | [.252, .293] | .000 |
| 16 | WB1 – WB3 | .252 | [.228, .274] | .000 |
| 17 | SM2 – SM4 | .251 | [.202, .333] | .000 |
| 18 | SM8 – SMP | .243 | [.209, .275] | .000 |
| 19 | SM3 – SM9 | .241 | [.158, .322] | .000 |
| 20 | SM1 – SM7 | .236 | [.165, .317] | .000 |
| 21 | SM5 – SM9 | .235 | [.151, .301] | .000 |
| 22 | SM6 – SM7 | .232 | [.154, .309] | .000 |
| 23 | WB3 – WB4 | .223 | [.201, .242] | .000 |
| 24 | eSK – AL | .214 | [.179, .249] | .000 |
| 25 | SM1 – SM5 | .212 | [.156, .291] | .000 |
| 26 | SM3 – SM6 | .204 | [.132, .281] | .000 |
| 27 | SM4 – SM7 | .203 | [.134, .267] | .000 |
| 28 | WB2 – WB4 | .196 | [.174, .216] | .000 |
| 29 | SK – AL | .189 | [.149, .224] | .000 |
| 30 | SM3 – SM5 | .188 | [.115, .259] | .000 |
| 31 | SM4 – SM6 | .182 | [.126, .253] | .000 |
| 32 | SM4 – SEX | .182 | [.141, .237] | .000 |
| 33 | SM2 – SM8 | .184 | [.120, .266] | .000 |
| 34 | WB4 – WB5 | .172 | [.151, .192] | .000 |
| 35 | SM1 – SM8 | .171 | [.111, .239] | .000 |
| 36 | SM8 – SM9 | .171 | [.088, .241] | .000 |
| 37 | FL – NV | .165 | [.143, .185] | .000 |
| 38 | FL – SEX | .160 | [.131, .188] | .000 |
| 39 | WB3 – WB5 | .159 | [.137, .180] | .000 |
| 40 | SM1 – SM9 | .157 | [.086, .240] | .001 |
| 41 | SMP – AGE | .156 | [.138, .177] | .000 |
| 42 | SM1 – SM4 | .156 | [.106, .223] | .000 |
| 43 | AL – AGE | .154 | [.137, .175] | .000 |
| 44 | SM8 – FL | .151 | [.117, .180] | .000 |
| 45 | SM7 – SM8 | .149 | [.072, .214] | .001 |
| 46 | SEX – AGE | −.148 | [−.186, −.119] | .000 |
| 47 | SM3 – SM8 | .146 | [.077, .209] | .000 |
| 48 | WB1 – WB5 | .146 | [.123, .167] | .000 |
| 49 | SM8 – SEX | .145 | [.100, .199] | .000 |
| 50 | SM8 – NV | .108 | [.072, .136] | .000 |
| 51 | SM5 – SM6 | .136 | [.067, .214] | .002 |
| 52 | SM5 – SM8 | .136 | [.065, .196] | .000 |
| 53 | WB3 – SEX | −.135 | [−.167, −.105] | .000 |
| 54 | WB3 – AGE | −.133 | [−.150, −.110] | .000 |
| 55 | SM1 – SM6 | .134 | [.070, .217] | .001 |
| 56 | SD – WB4 | −.126 | [−.146, −.107] | .000 |
| 57 | NV – SD | .126 | [.105, .147] | .000 |
| 58 | SM2 – SM9 | .125 | [.044, .212] | .007 |
| 59 | IR – SD | .123 | [.100, .143] | .000 |
| 60 | WB2 – WB5 | .123 | [.100, .144] | .000 |
| 61 | SM1 – SMP | .121 | [.090, .166] | .000 |
| 62 | SM2 – SM5 | .120 | [.051, .206] | .005 |
| 63 | FL – SD | .114 | [.090, .133] | .000 |
| 64 | NV – SEX | .111 | [.083, .140] | .000 |
| 65 | IR – WB1 | −.109 | [−.127, −.087] | .000 |
| 66 | SM2 – SM7 | .104 | [.025, .189] | .017 |
| 67 | SM1 – NV | .030 | [.000, .064] | .262 |
| 68 | SD – AGE | −.099 | [−.121, −.077] | .000 |
| 69 | SM8 – AGE | .095 | [.061, .127] | .000 |
| 70 | NV – WB2 | −.094 | [−.113, −.074] | .000 |
| 71 | WB2 – WB3 | .086 | [.063, .109] | .000 |
| 72 | FL – AGE | .085 | [.065, .104] | .000 |
| 73 | SMP – FL | .082 | [.059, .101] | .000 |
| 74 | FL – WB1 | −.081 | [−.102, −.059] | .000 |
| 75 | WB2 – AGE | .073 | [.051, .092] | .000 |
| 76 | SM4 – AGE | −.069 | [−.098, −.034] | .014 |
| 77 | WB2 – SEX | −.068 | [−.102, −.043] | .000 |
| 78 | SM6 – IR | .064 | [.028, .103] | .012 |
| 79 | SM7 – SD | .063 | [.000, .104] | .045 |
| 80 | eSK – AGE | .060 | [.040, .075] | .001 |
| 81 | WB4 – AGE | −.059 | [−.079, −.038] | .000 |
| 82 | FL – WB3 | −.059 | [−.076, −.038] | .000 |
| 83 | SM9 – eSK | .054 | [.000, .090] | .158 |
| 84 | SM7 – AL | .054 | [.000, .095] | .358 |
| 85 | IR – WB4 | −.051 | [−.071, −.030] | .000 |
| 86 | SM7 – AGE | −.051 | [−.093, .000] | .618 |
| 87 | SM9 – IR | .050 | [.000, .085] | .342 |
| 88 | AL – FAS | .046 | [.023, .069] | .010 |
| 89 | SM3 – SK | .045 | [.000, .076] | .267 |
| 90 | SM5 – WB3 | −.045 | [−.074, .000] | .356 |
| 91 | SM9 – AL | .043 | [.000, .089] | .403 |
| 92 | AL – SEX | −.042 | [−.087, −.017] | .023 |
| 93 | WB1 – SEX | .042 | [.000, .095] | .213 |
| 94 | SM4 – SM9 | .040 | [.000, .107] | .330 |
| 95 | IR – WB2 | −.040 | [−.061, −.017] | .006 |
| 96 | SD – SK | .039 | [.000, .054] | .075 |
| 97 | SM8 – IR | .037 | [.000, .067] | .088 |
| 98 | SM7 – WB5 | −.034 | [−.077, .000] | .311 |
| 99 | SD – WB1 | −.033 | [−.052, .000] | .026 |
| 100 | SM8 – WB1 | −.033 | [−.064, .000] | .492 |
| 101 | FL – eSK | .032 | [.011, .046] | .021 |
| 102 | SM3 – SD | .029 | [.000, .065] | .518 |
| 103 | SK – FAS | −.029 | [−.048, .000] | .223 |
| 104 | SMP – WB3 | −.027 | [−.043, .000] | .156 |
| 105 | SM8 – WB4 | −.026 | [−.055, .000] | .222 |
| 106 | SM3 – IR | .026 | [.000, .056] | .482 |
| 107 | WB4 – SEX | −.024 | [−.057, .000] | .327 |
| 108 | SM8 – WB3 | −.024 | [−.055, .000] | .404 |
| 109 | SMP – IR | .024 | [.000, .041] | .466 |
| 110 | FL – WB2 | −.022 | [−.043, .000] | .152 |
| 111 | IR – WB5 | −.022 | [−.038, .000] | .158 |
| 112 | SM7 – NV | .021 | [.000, .062] | .529 |
| 113 | NV – WB4 | −.020 | [−.040, .000] | .295 |
| 114 | IR – AL | .020 | [.000, .038] | .397 |
| 115 | WB4 – AL | −.019 | [−.040, .000] | .134 |
| 116 | SM8 – WB5 | −.018 | [−.050, .000] | .414 |
| 117 | WB1 – WB4 | .017 | [.000, .039] | .330 |
| 118 | SM4 – SD | .016 | [.000, .045] | .507 |
| 119 | SD – AL | .015 | [.000, .038] | .242 |
| 120 | FL – WB4 | −.013 | [−.033, .000] | .499 |
| 121 | SM4 – WB4 | −.013 | [−.040, .000] | .622 |
| 122 | SMP – AL | .012 | [.000, .035] | .397 |
| 123 | SMP – SD | .012 | [.000, .034] | .358 |
| 124 | SM5 – AL | .011 | [.000, .049] | .625 |
| 125 | WB5 – eSK | −.010 | [−.027, .000] | .628 |
| 126 | AGE – FAS | .008 | [.000, .028] | .626 |
