# Supplementary material for: Problematic social media use, mental health, and substance use in Czech adolescents: the comparative role of extracurricular activities — findings from the 2022 HBSC study
Source: Arch Public Health. 2026 Jun 16;84:192. doi: 10.1186/s13690-026-01993-1 (PMC13508371; doi:10.1186/s13690-026-01993-1)
Supplement: Supplementary file 8 [file 13690_2026_1993_MOESM5_ESM.docx]

|  | **SM1** | **SM2** | **SM3** | **SM4** | **SM5** | **SM6** | **SM7** | **SM8** | **SM9** | **SMP** | **FL** | **IR** | **NV** | **SD** | **WB1** | **WB2** | **WB3** | **WB4** | **WB5** | **SK** | **eSK** | **AL** | **SEX** | **ART** | **AGE** | **FAS** |
| --- | --- | --- | --- | --- | --- | --- | --- | --- | --- | --- | --- | --- | --- | --- | --- | --- | --- | --- | --- | --- | --- | --- | --- | --- | --- | --- |
| SM1 | — | .636 | .659 | .383 | .446 | .476 | .517 | .432 | .495 | .274 | .219 | .264 | .254 | .226 | −.176 | −.175 | −.154 | −.197 | −.172 | .209 | .226 | .166 | .125 | −.026 | .001 | −.002 |
| SM2 |  | — | .666 | .427 | .430 | .534 | .493 | .447 | .498 | .230 | .214 | .276 | .248 | .225 | −.212 | −.200 | −.165 | −.209 | −.194 | .261 | .219 | .175 | .077 | −.017 | −.002 | −.024 |
| SM3 |  |  | — | .331 | .448 | .510 | .554 | .424 | .536 | .235 | .236 | .278 | .250 | .248 | −.180 | −.186 | −.153 | −.192 | −.183 | .289 | .253 | .192 | .123 | −.027 | .010 | .002 |
| SM4 |  |  |  | — | .391 | .386 | .396 | .436 | .345 | .163 | .199 | .214 | .197 | .192 | −.147 | −.152 | −.145 | −.171 | −.142 | .101 | .113 | .075 | .243 | .069 | −.039 | −.028 |
| SM5 |  |  |  |  | — | .415 | .492 | .366 | .462 | .172 | .210 | .207 | .196 | .168 | −.177 | −.150 | −.180 | −.165 | −.159 | .219 | .193 | .158 | .104 | −.005 | .031 | −.041 |
| SM6 |  |  |  |  |  | — | .518 | .456 | .631 | .222 | .221 | .289 | .230 | .223 | −.166 | −.161 | −.122 | −.160 | −.140 | .251 | .237 | .184 | .072 | .018 | .005 | −.011 |
| SM7 |  |  |  |  |  |  | — | .421 | .623 | .211 | .237 | .267 | .253 | .261 | −.207 | −.171 | −.143 | −.196 | −.199 | .221 | .213 | .193 | .104 | .034 | −.015 | .010 |
| SM8 |  |  |  |  |  |  |  | — | .438 | .399 | .485 | .442 | .418 | .326 | −.363 | −.318 | −.339 | −.339 | −.290 | .231 | .256 | .225 | .307 | .075 | .163 | −.049 |
| SM9 |  |  |  |  |  |  |  |  | — | .203 | .271 | .315 | .271 | .261 | −.212 | −.197 | −.150 | −.207 | −.192 | .274 | .283 | .216 | .129 | .052 | .005 | −.005 |
| SMP |  |  |  |  |  |  |  |  |  | — | .331 | .285 | .251 | .205 | −.209 | −.177 | −.239 | −.213 | −.185 | .177 | .225 | .216 | .142 | −.001 | .251 | .004 |
| FL |  |  |  |  |  |  |  |  |  |  | — | .727 | .607 | .487 | −.520 | −.477 | −.466 | −.470 | −.364 | .326 | .324 | .254 | .381 | .136 | .195 | −.022 |
| IR |  |  |  |  |  |  |  |  |  |  |  | — | .635 | .509 | −.537 | −.491 | −.438 | −.469 | −.385 | .287 | .297 | .234 | .296 | .094 | .118 | −.021 |
| NV |  |  |  |  |  |  |  |  |  |  |  |  | — | .473 | −.402 | −.441 | −.358 | −.400 | −.295 | .192 | .197 | .138 | .339 | .099 | .098 | −.013 |
| SD |  |  |  |  |  |  |  |  |  |  |  |  |  | — | −.379 | −.348 | −.316 | −.422 | −.274 | .269 | .230 | .188 | .233 | .087 | .000 | −.013 |
| WB1 |  |  |  |  |  |  |  |  |  |  |  |  |  |  | — | .690 | .626 | .548 | .544 | −.183 | −.165 | −.152 | −.220 | −.040 | −.091 | .074 |
| WB2 |  |  |  |  |  |  |  |  |  |  |  |  |  |  |  | — | .546 | .576 | .515 | −.137 | −.141 | −.098 | −.295 | −.074 | −.035 | .070 |
| WB3 |  |  |  |  |  |  |  |  |  |  |  |  |  |  |  |  | — | .586 | .521 | −.212 | −.212 | −.181 | −.304 | −.059 | −.212 | .072 |
| WB4 |  |  |  |  |  |  |  |  |  |  |  |  |  |  |  |  |  | — | .516 | −.235 | −.226 | −.220 | −.270 | −.063 | −.152 | .037 |
| WB5 |  |  |  |  |  |  |  |  |  |  |  |  |  |  |  |  |  |  | — | −.208 | −.204 | −.140 | −.213 | .005 | −.099 | .075 |
| SK |  |  |  |  |  |  |  |  |  |  |  |  |  |  |  |  |  |  |  | — | .829 | .672 | .064 | −.110 | .376 | −.041 |
| eSK |  |  |  |  |  |  |  |  |  |  |  |  |  |  |  |  |  |  |  |  | — | .639 | .088 | −.088 | .405 | .018 |
| AL |  |  |  |  |  |  |  |  |  |  |  |  |  |  |  |  |  |  |  |  |  | — | −.017 | −.053 | .413 | .085 |
| SEX |  |  |  |  |  |  |  |  |  |  |  |  |  |  |  |  |  |  |  |  |  |  | — | .447 | −.059 | −.047 |
| ART |  |  |  |  |  |  |  |  |  |  |  |  |  |  |  |  |  |  |  |  |  |  |  | — | −.138 | .084 |
| AGE |  |  |  |  |  |  |  |  |  |  |  |  |  |  |  |  |  |  |  |  |  |  |  |  | — | .026 |
| FAS |  |  |  |  |  |  |  |  |  |  |  |  |  |  |  |  |  |  |  |  |  |  |  |  |  | — |

**Edge Weight Matrix**

|  | **SM1** | **SM2** | **SM3** | **SM4** | **SM5** | **SM6** | **SM7** | **SM8** | **SM9** | **SMP** | **FL** | **IR** | **NV** | **SD** | **WB1** | **WB2** | **WB3** | **WB4** | **WB5** | **SK** | **eSK** | **AL** | **SEX** | **ART** | **AGE** | **FAS** |
| --- | --- | --- | --- | --- | --- | --- | --- | --- | --- | --- | --- | --- | --- | --- | --- | --- | --- | --- | --- | --- | --- | --- | --- | --- | --- | --- |
| SM1 | — | .511 | .607 | .164 | .217 | .137 | .242 | .171 | .164 | .131 |  |  | .033 |  |  |  |  |  |  |  |  |  |  |  |  |  |
| SM2 |  | — | .604 | .253 | .118 | .303 | .107 | .182 | .123 |  |  |  |  |  |  |  |  |  |  |  |  |  |  |  |  |  |
| SM3 |  |  | — |  | .187 | .202 | .333 | .143 | .240 |  |  | .023 |  | .031 |  |  |  |  |  | .035 |  |  |  |  |  |  |
| SM4 |  |  |  | — | .278 | .183 | .204 | .359 | .037 |  |  |  |  | .017 |  |  |  |  |  |  |  |  | .197 |  | .070 |  |
| SM5 |  |  |  |  | — | .136 | .333 | .133 | .234 |  |  |  |  |  |  |  | .045 |  |  |  |  |  |  |  |  |  |
| SM6 |  |  |  |  |  | — | .231 | .274 | .612 |  |  | .064 |  |  |  |  |  |  |  |  |  |  |  |  |  |  |
| SM7 |  |  |  |  |  |  | — | .147 | .563 |  |  |  | .020 | .066 |  |  |  |  | .034 |  |  | .054 |  |  |  |  |
| SM8 |  |  |  |  |  |  |  | — | .167 | .245 | .146 | .038 | .106 |  | .032 |  | .025 | .027 | .015 |  |  |  | .152 |  | .093 | .016 |
| SM9 |  |  |  |  |  |  |  |  | — |  |  | .049 |  |  |  |  |  |  |  |  | .055 | .045 |  |  |  |  |
| SMP |  |  |  |  |  |  |  |  |  | — | .081 | .023 |  | .014 |  |  | .027 |  |  |  |  | .017 |  |  | .157 |  |
| FL |  |  |  |  |  |  |  |  |  |  | — | .364 | .164 | .113 | .081 | .022 | .058 |  |  |  | .031 |  | .149 | .026 | .085 |  |
| IR |  |  |  |  |  |  |  |  |  |  |  | — | .275 | .123 | .109 | .040 |  | .053 | .021 |  |  | .021 |  |  |  |  |
| NV |  |  |  |  |  |  |  |  |  |  |  |  | — | .126 |  | .095 |  | .018 |  |  |  |  | .116 |  |  |  |
| SD |  |  |  |  |  |  |  |  |  |  |  |  |  | — | .034 |  |  | .125 |  | .036 |  | .018 |  |  | .098 |  |
| WB1 |  |  |  |  |  |  |  |  |  |  |  |  |  |  | — | .352 | .252 | .016 | .146 |  |  |  | .062 |  |  |  |
| WB2 |  |  |  |  |  |  |  |  |  |  |  |  |  |  |  | — | .086 | .195 | .123 |  |  | .011 | .075 |  | .072 |  |
| WB3 |  |  |  |  |  |  |  |  |  |  |  |  |  |  |  |  | — | .223 | .159 |  |  |  | .140 |  | .131 |  |
| WB4 |  |  |  |  |  |  |  |  |  |  |  |  |  |  |  |  |  | — | .171 |  |  | .022 | .022 |  | .055 |  |
| WB5 |  |  |  |  |  |  |  |  |  |  |  |  |  |  |  |  |  |  | — |  | .010 |  |  |  |  |  |
| SK |  |  |  |  |  |  |  |  |  |  |  |  |  |  |  |  |  |  |  | — | .491 | .188 |  |  |  | .028 |
| eSK |  |  |  |  |  |  |  |  |  |  |  |  |  |  |  |  |  |  |  |  | — | .213 |  |  | .059 |  |
| AL |  |  |  |  |  |  |  |  |  |  |  |  |  |  |  |  |  |  |  |  |  | — | .067 |  | .157 | .055 |
| SEX |  |  |  |  |  |  |  |  |  |  |  |  |  |  |  |  |  |  |  |  |  |  | — | .445 | .109 | .038 |
| ART |  |  |  |  |  |  |  |  |  |  |  |  |  |  |  |  |  |  |  |  |  |  |  | — | .093 | .065 |
| AGE |  |  |  |  |  |  |  |  |  |  |  |  |  |  |  |  |  |  |  |  |  |  |  |  | — | .020 |
| FAS |  |  |  |  |  |  |  |  |  |  |  |  |  |  |  |  |  |  |  |  |  |  |  |  |  | — |

**Bootstrapped 95% Confidence Intervals for Non-Zero Edge Weights**

| **Rank** | **Edge** | **Weight** | **95% CI** | **Prop. zero** |
| --- | --- | --- | --- | --- |
| 1 | SM6 – SM9 | .612 | [.541, .694] | .000 |
| 2 | SM1 – SM3 | .607 | [.547, .675] | .000 |
| 3 | SM2 – SM3 | .604 | [.540, .681] | .000 |
| 4 | SM7 – SM9 | .563 | [.485, .635] | .000 |
| 5 | SM1 – SM2 | .511 | [.451, .588] | .000 |
| 6 | SK – eSK | .491 | [.448, .527] | .000 |
| 7 | SEX – ART | .445 | [.408, .473] | .000 |
| 8 | FL – IR | .364 | [.344, .382] | .000 |
| 9 | SM4 – SM8 | .359 | [.308, .405] | .000 |
| 10 | WB1 – WB2 | .352 | [.331, .371] | .000 |
| 11 | SM3 – SM7 | .333 | [.255, .408] | .000 |
| 12 | SM5 – SM7 | .333 | [.254, .398] | .000 |
| 13 | SM2 – SM6 | .303 | [.233, .387] | .000 |
| 14 | SM4 – SM5 | .278 | [.211, .331] | .000 |
| 15 | IR – NV | .275 | [.253, .294] | .000 |
| 16 | SM6 – SM8 | .274 | [.213, .354] | .000 |
| 17 | SM2 – SM4 | .253 | [.200, .333] | .000 |
| 18 | WB1 – WB3 | .252 | [.229, .273] | .000 |
| 19 | SM8 – SMP | .245 | [.211, .278] | .000 |
| 20 | SM1 – SM7 | .242 | [.167, .317] | .000 |
| 21 | SM3 – SM9 | .240 | [.158, .319] | .000 |
| 22 | SM5 – SM9 | .234 | [.154, .304] | .000 |
| 23 | SM6 – SM7 | .231 | [.148, .311] | .000 |
| 24 | WB3 – WB4 | .223 | [.201, .241] | .000 |
| 25 | SM1 – SM5 | .217 | [.146, .285] | .000 |
| 26 | eSK – AL | .213 | [.177, .246] | .000 |
| 27 | SM3 – SM6 | .202 | [.131, .284] | .000 |
| 28 | SM4 – SM7 | .204 | [.130, .271] | .000 |
| 29 | SM4 – SEX | .197 | [.140, .238] | .000 |
| 30 | WB2 – WB4 | .195 | [.174, .216] | .000 |
| 31 | SM2 – SM8 | .182 | [.119, .264] | .000 |
| 32 | SK – AL | .188 | [.150, .224] | .000 |
| 33 | SM3 – SM5 | .187 | [.113, .259] | .000 |
| 34 | SM4 – SM6 | .183 | [.126, .258] | .000 |
| 35 | SM1 – SM8 | .171 | [.108, .239] | .000 |
| 36 | WB4 – WB5 | .171 | [.152, .192] | .000 |
| 37 | SM8 – SM9 | .167 | [.092, .241] | .000 |
| 38 | SM1 – SM4 | .164 | [.107, .227] | .000 |
| 39 | SM1 – SM9 | .164 | [.088, .242] | .000 |
| 40 | FL – NV | .164 | [.142, .185] | .000 |
| 41 | WB3 – WB5 | .159 | [.137, .180] | .000 |
| 42 | SMP – AGE | .157 | [.136, .174] | .000 |
| 43 | AL – AGE | .157 | [.137, .175] | .000 |
| 44 | SM8 – SEX | .152 | [.099, .200] | .000 |
| 45 | FL – SEX | .149 | [.117, .177] | .000 |
| 46 | SM8 – FL | .146 | [.116, .179] | .000 |
| 47 | WB1 – WB5 | .146 | [.124, .166] | .000 |
| 48 | SM7 – SM8 | .147 | [.067, .218] | .001 |
| 49 | SM3 – SM8 | .143 | [.073, .214] | .000 |
| 50 | SM1 – SM6 | .137 | [.068, .212] | .000 |
| 51 | SM5 – SM6 | .136 | [.065, .214] | .000 |
| 52 | WB3 – SEX | −.140 | [−.165, −.102] | .000 |
| 53 | SM5 – SM8 | .133 | [.069, .197] | .000 |
| 54 | WB3 – AGE | −.131 | [−.150, −.109] | .000 |
| 55 | SD – WB4 | −.125 | [−.145, −.107] | .000 |
| 56 | NV – SD | .126 | [.105, .147] | .000 |
| 57 | SM2 – SM5 | .118 | [.052, .201] | .004 |
| 58 | SM2 – SM9 | .123 | [.042, .209] | .010 |
| 59 | IR – SD | .123 | [.099, .142] | .000 |
| 60 | WB2 – WB5 | .123 | [.101, .145] | .000 |
| 61 | SM1 – SMP | .131 | [.091, .169] | .000 |
| 62 | FL – SD | .113 | [.090, .134] | .000 |
| 63 | NV – SEX | .116 | [.085, .143] | .000 |
| 64 | IR – WB1 | −.109 | [−.128, −.087] | .000 |
| 65 | SM8 – NV | .106 | [.074, .136] | .000 |
| 66 | SEX – AGE | −.109 | [−.141, −.076] | .000 |
| 67 | SM2 – SM7 | .107 | [.000, .189] | .026 |
| 68 | SM8 – AGE | .092 | [.062, .130] | .000 |
| 69 | ART – AGE | −.093 | [−.111, −.072] | .000 |
| 70 | SD – AGE | −.098 | [−.119, −.075] | .000 |
| 71 | NV – WB2 | −.095 | [−.112, −.075] | .000 |
| 72 | WB2 – WB3 | .086 | [.061, .107] | .000 |
| 73 | FL – AGE | .085 | [.067, .106] | .000 |
| 74 | SMP – FL | .081 | [.060, .102] | .000 |
| 75 | FL – WB1 | −.081 | [−.103, −.060] | .000 |
| 76 | WB2 – SEX | −.075 | [−.103, −.042] | .000 |
| 77 | WB2 – AGE | .072 | [.050, .092] | .000 |
| 78 | SM4 – AGE | −.070 | [−.100, −.039] | .009 |
| 79 | AL – SEX | −.067 | [−.093, −.017] | .024 |
| 80 | SM7 – SD | .066 | [.000, .104] | .039 |
| 81 | ART – FAS | .065 | [.040, .083] | .000 |
| 82 | SM6 – IR | .064 | [.027, .102] | .012 |
| 83 | WB1 – SEX | .062 | [.000, .096] | .241 |
| 84 | eSK – AGE | .059 | [.040, .074] | .002 |
| 85 | WB4 – AGE | −.055 | [−.079, −.037] | .000 |
| 86 | AL – FAS | .055 | [.026, .071] | .003 |
| 87 | SM9 – eSK | .055 | [.000, .087] | .179 |
| 88 | SM7 – AL | .054 | [.000, .093] | .461 |
| 89 | IR – WB4 | −.053 | [−.072, −.031] | .000 |
| 90 | FL – WB3 | −.058 | [−.076, −.037] | .000 |
| 91 | SM9 – IR | .049 | [.000, .084] | .365 |
| 92 | SM9 – AL | .045 | [.000, .087] | .490 |
| 93 | SM5 – WB3 | −.045 | [−.074, .000] | .383 |
| 94 | SM3 – SK | .035 | [.000, .072] | .282 |
| 95 | SM8 – IR | .038 | [.000, .068] | .069 |
| 96 | SM7 – WB5 | −.034 | [−.076, .000] | .360 |
| 97 | SM1 – NV | .033 | [.000, .065] | .250 |
| 98 | SD – WB1 | −.034 | [−.053, .000] | .029 |
| 99 | SM3 – SD | .031 | [.000, .069] | .488 |
| 100 | FL – eSK | .031 | [.000, .046] | .025 |
| 101 | SD – SK | .036 | [.000, .054] | .101 |
| 102 | SK – FAS | −.028 | [−.049, .000] | .196 |
| 103 | SMP – WB3 | −.027 | [−.042, .000] | .175 |
| 104 | FL – ART | .026 | [.000, .042] | .255 |
| 105 | SM8 – WB4 | −.027 | [−.057, .000] | .207 |
| 106 | SM8 – WB3 | −.025 | [−.055, .000] | .401 |
| 107 | SM3 – IR | .023 | [.000, .053] | .526 |
| 108 | SMP – IR | .023 | [.000, .040] | .505 |
| 109 | WB4 – AL | −.022 | [−.040, .000] | .159 |
| 110 | WB4 – SEX | −.022 | [−.055, .000] | .360 |
| 111 | FL – WB2 | −.022 | [−.042, .000] | .160 |
| 112 | IR – WB5 | −.021 | [−.037, .000] | .188 |
| 113 | IR – AL | .021 | [.000, .037] | .415 |
| 114 | SM7 – NV | .020 | [.000, .058] | .558 |
| 115 | AGE – FAS | .020 | [.000, .037] | .269 |
| 116 | NV – WB4 | −.018 | [−.038, .000] | .335 |
| 117 | SD – AL | .018 | [.000, .037] | .267 |
| 118 | SM4 – SD | .017 | [.000, .044] | .503 |
| 119 | SMP – AL | .017 | [.000, .036] | .383 |
| 120 | SM8 – FAS | −.016 | [−.053, .000] | .494 |
| 121 | SM8 – WB5 | −.015 | [−.048, .000] | .484 |
| 122 | SM8 – WB1 | −.032 | [−.063, .000] | .528 |
| 123 | SMP – SD | .013 | [.000, .032] | .404 |
| 124 | WB2 – AL | .011 | [.000, .030] | .710 |
| 125 | WB5 – eSK | −.010 | [−.028, .000] | .650 |
| 127 | FL – WB4 | .000 | [−.033, .000] | .541 |
| 128 | SM4 – WB4 | .000 | [−.037, .000] | .698 |
| 129 | IR – WB2 | −.040 | [−.060, −.017] | .003 |
