# Supplementary material for: Problematic social media use, mental health, and substance use in Czech adolescents: the comparative role of extracurricular activities — findings from the 2022 HBSC study
Source: Arch Public Health. 2026 Jun 16;84:192. doi: 10.1186/s13690-026-01993-1 (PMC13508371; doi:10.1186/s13690-026-01993-1)
Supplement: Supplementary file 9 [file 13690_2026_1993_MOESM6_ESM.docx]

| **Node** | **Variable type** | **Metric** | **Value** | **Baseline** | **Normalized CC** |
| --- | --- | --- | --- | --- | --- |
| **Panel A: Social media use (categorical)** |  |  |  |  |  |
| SM1 | Categorical | CC | .832 | .771 | .266 |
| SM2 | Categorical | CC | .866 | .822 | .247 |
| SM3 | Categorical | CC | .858 | .804 | .276 |
| SM4 | Categorical | CC | .711 | .649 | .177 |
| SM5 | Categorical | CC | .834 | .814 | .108 |
| SM6 | Categorical | CC | .855 | .829 | .152 |
| SM7 | Categorical | CC | .872 | .849 | .152 |
| SM8 | Categorical | CC | .740 | .556 | .414 |
| SM9 | Categorical | CC | .879 | .851 | .188 |
| **Panel B: Mental health complaints (Gaussian)** |  |  |  |  |  |
| SMP | Gaussian | R² | .172 | — | — |
| FL | Gaussian | R² | .537 | — | — |
| IR | Gaussian | R² | .555 | — | — |
| NV | Gaussian | R² | .422 | — | — |
| SD | Gaussian | R² | .287 | — | — |
| **Panel C: Wellbeing (Gaussian)** |  |  |  |  |  |
| WB1 | Gaussian | R² | .532 | — | — |
| WB2 | Gaussian | R² | .510 | — | — |
| WB3 | Gaussian | R² | .478 | — | — |
| WB4 | Gaussian | R² | .441 | — | — |
| WB5 | Gaussian | R² | .356 | — | — |
| **Panel D: Substance use (Gaussian)** |  |  |  |  |  |
| SK | Gaussian | R² | .391 | — | — |
| eSK | Gaussian | R² | .408 | — | — |
| AL | Gaussian | R² | .263 | — | — |
| **Panel E: Covariates** |  |  |  |  |  |
| SEX | Categorical | CC | .659 | .507 | .308 |
| AGE | Gaussian | R² | .182 | — | — |
| FAS | Gaussian | R² | .014 | — | — |

The nodes with the highest predictability were irritability (IR; R² = .555), feeling low (FL; R² = .537), and felt cheerful (WB1; R² = .532). These values indicate that over half of the variance in these nodes was accounted for by their neighboring nodes in the network. This suggests that these mental health complaint and wellbeing nodes are deeply embedded in the network structure — their levels are closely tied to the other variables in the system. Nervousness (NV; R² = .422) and the remaining wellbeing nodes (WB2–WB5; R² = .356–.510) also showed moderate to moderately high predictability, reinforcing the interpretation that these domains are well-interconnected within the network. The raw classification accuracy for the PSMU nodes ranged from .711 (SM4) to .879 (SM9), which may appear high at first glance. However, these values must be interpreted relative to the baseline accuracy (the proportion of the majority class). Because most PSMU items had highly skewed distributions, with the majority of participants endorsing the "no" category (baselines ranging from .556 to .851), much of the raw CC simply reflects this class imbalance rather than genuine predictive power from the network. The normalized CC provides a more meaningful picture. SM8 ("using social media to escape negative feelings") had the highest normalized accuracy (.414), indicating that its neighbors explained a substantial proportion of its variance beyond chance. This is consistent with SM8's strong edges to FL, NV, and IR observed in the network graph. SM3 ("feeling bad when not using social media"; normalized CC = .276), SM1 ("can't think of anything else"; .266), and SM2 ("felt dissatisfied"; .247) also showed meaningful predictability above baseline. In contrast, SM5 ("lost interest in other activities"; normalized CC = .108) had the lowest normalized accuracy among the PSMU nodes, suggesting that despite its many connections in the network, its categorical status was only minimally predicted by neighboring nodes. This could indicate that the factors determining whether an adolescent endorses this item lie largely outside the current network. Cigarette smoking (SK; R² = .391) and e-cigarette smoking (eSK; R² = .408) showed moderate predictability, largely driven by their strong mutual edge (SK–eSK) rather than extensive connections to the rest of the network. Alcohol use (AL; R² = .263) was less well predicted, suggesting it is more peripherally connected.
