# Supplementary material for: Problematic social media use, mental health, and substance use in Czech adolescents: the comparative role of extracurricular activities — findings from the 2022 HBSC study
Source: Arch Public Health. 2026 Jun 16;84:192. doi: 10.1186/s13690-026-01993-1 (PMC13508371; doi:10.1186/s13690-026-01993-1)
Supplement: Supplementary file 10 [file 13690_2026_1993_MOESM7_ESM.docx]

| **Edge** | **Weight (γ = 0.50)** | **Weight (γ = 0.75)** | **Δ Weight** | **Status** |
| --- | --- | --- | --- | --- |
| **Panel A: Edges removed at γ = 0.75** |  |  |  |  |
| SM7 – AL | 0.054 | 0.000 | −0.054 | Removed |
| SM7 – AGE | 0.051 | 0.000 | −0.051 | Removed |
| SM9 – IR | 0.050 | 0.000 | −0.050 | Removed |
| SM3 – IR | 0.026 | 0.000 | −0.026 | Removed |
| SMP – IR | 0.024 | 0.000 | −0.024 | Removed |
| IR – AL | 0.020 | 0.000 | −0.020 | Removed |
| FL – WB4 | 0.013 | 0.000 | −0.013 | Removed |
| SM5 – AL | 0.011 | 0.000 | −0.011 | Removed |
| AGE – FAS | 0.008 | 0.000 | −0.008 | Removed |
| **Panel B: Edges with changed weights** |  |  |  |  |
| SM7 – SM9 | 0.567 | 0.564 | −0.004 | Changed |
| FL – IR | 0.364 | 0.365 | +0.001 | Changed |
| SM3 – SM7 | 0.337 | 0.333 | −0.004 | Changed |
| SM5 – SM7 | 0.331 | 0.312 | −0.019 | Changed |
| SM4 – SM5 | 0.275 | 0.256 | −0.019 | Changed |
| IR – NV | 0.274 | 0.270 | −0.004 | Changed |
| SM1 – SM7 | 0.236 | 0.233 | −0.003 | Changed |
| SM5 – SM9 | 0.235 | 0.227 | −0.009 | Changed |
| SM6 – SM7 | 0.232 | 0.227 | −0.005 | Changed |
| SM1 – SM5 | 0.212 | 0.203 | −0.009 | Changed |
| SM4 – SM7 | 0.203 | 0.194 | −0.009 | Changed |
| SM3 – SM5 | 0.188 | 0.180 | −0.008 | Changed |
| FL – NV | 0.165 | 0.164 | −0.001 | Changed |
| FL – SEX | 0.160 | 0.156 | −0.005 | Changed |
| SM8 – FL | 0.151 | 0.147 | −0.004 | Changed |
| SM7 – SM8 | 0.149 | 0.142 | −0.007 | Changed |
| SM5 – SM6 | 0.136 | 0.125 | −0.010 | Changed |
| SM5 – SM8 | 0.136 | 0.125 | −0.011 | Changed |
| IR – SD | 0.123 | 0.118 | −0.005 | Changed |
| SM2 – SM5 | 0.120 | 0.113 | −0.006 | Changed |
| FL – SD | 0.114 | 0.112 | −0.002 | Changed |
| IR – WB1 | 0.109 | 0.107 | −0.002 | Changed |
| SM2 – SM7 | 0.104 | 0.102 | −0.002 | Changed |
| FL – AGE | 0.085 | 0.083 | −0.002 | Changed |
| SMP – FL | 0.082 | 0.080 | −0.002 | Changed |
| FL – WB1 | 0.081 | 0.081 | −0.001 | Changed |
| SM6 – IR | 0.064 | 0.046 | −0.018 | Changed |
| SM7 – SD | 0.063 | 0.061 | −0.003 | Changed |
| FL – WB3 | 0.059 | 0.059 | 0.000 | Changed |
| IR – WB4 | 0.051 | 0.049 | −0.002 | Changed |
| AL – FAS | 0.046 | 0.042 | −0.004 | Changed |
| SM5 – WB3 | 0.045 | 0.035 | −0.010 | Changed |
| IR – WB2 | 0.040 | 0.037 | −0.004 | Changed |
| SM8 – IR | 0.037 | 0.031 | −0.006 | Changed |
| SM7 – WB5 | 0.034 | 0.029 | −0.006 | Changed |
| FL – eSK | 0.032 | 0.031 | −0.001 | Changed |
| SK – FAS | 0.029 | 0.025 | −0.004 | Changed |
| FL – WB2 | 0.022 | 0.021 | −0.001 | Changed |
| IR – WB5 | 0.022 | 0.016 | −0.006 | Changed |
| SM7 – NV | 0.021 | 0.017 | −0.004 | Changed |
